# A novel feature selection algorithm based on damping oscillation theory

**DOI:** 10.1371/journal.pone.0255307

**Published:** 2021-08-06

**Authors:** Fujun Wang, Xing Wang

**Affiliations:** 1 School of Electronic and Information Engineering, Liaoning Technical University, Huludao, People’s Republic of China; 2 Key Laboratory of Preparation and Application of Environmentally Friendly Materials, Chinese Ministry of Education, Jilin Normal University, Changchun, People’s Republic of China; Torrens University Australia, AUSTRALIA

## Abstract

Feature selection is an important task in big data analysis and information retrieval processing. It reduces the number of features by removing noise, extraneous data. In this paper, one feature subset selection algorithm based on damping oscillation theory and support vector machine classifier is proposed. This algorithm is called the Maximum Kendall coefficient Maximum Euclidean Distance Improved Gray Wolf Optimization algorithm (MKMDIGWO). In MKMDIGWO, first, a filter model based on Kendall coefficient and Euclidean distance is proposed, which is used to measure the correlation and redundancy of the candidate feature subset. Second, the wrapper model is an improved grey wolf optimization algorithm, in which its position update formula has been improved in order to achieve optimal results. Third, the filter model and the wrapper model are dynamically adjusted by the damping oscillation theory to achieve the effect of finding an optimal feature subset. Therefore, MKMDIGWO achieves both the efficiency of the filter model and the high precision of the wrapper model. Experimental results on five UCI public data sets and two microarray data sets have demonstrated the higher classification accuracy of the MKMDIGWO algorithm than that of other four state-of-the-art algorithms. The maximum ACC value of the MKMDIGWO algorithm is at least 0.5% higher than other algorithms on 10 data sets.

## Introduction

In recent years, many irrelevant and redundant data have been discovered in data collection and sorting. In order to reduce the complexity of calculation and improve the accuracy of data analysis, and to reduce the storage space at the same time, the feature selection method has become the primary data processing method for researchers [1–3].

The two basic types of feature selection methods are filter and wrapper. The filter method has a low time complexity in the process of searching the optimal feature subset [4,5], which has been well demonstrated by the famous MRMR algorithm [6], GRM algorithm [3], ReliefF and tuned ReliefF algorithm [7].

On the other hand, the wrapper method generally achieves higher classification accuracy. In recent years, the wrapper method has gained more and more attention in feature selection [8]. The wrapper method consists of two parts: Meta-heuristic algorithms and classifier. Meta-heuristic algorithms play an important role in the wrapper method. Common meta-heuristic algorithms are, Ant Lion Optimization (ALO) [9], Bat Algorithm (BA) [10,11], Bacteria Foraging Optimization (BFO) [12], Cuckoo Search(CS) [13,14], Genetic Algorithm(GA) [15,16], Particle Swarm Optimization(PSO) [17,18] and Simulated Annealing(SA) [19,20]. The seven algorithms mentioned above are Swarm Intelligence algorithms, which are often used in wrapper feature selection algorithms. In the wrapper method, the commonly used classifiers are Support Vector Machine (SVM), Random Forest (RF), etc.

Algorithms included wrapper method and filter method are often entitled hybrid algorithms. Chen et al. combined the PSO algorithm with the SSA algorithm to propose the HPSO-SSM algorithm [21]. Mafarja et al. combined the WOA and SA algorithms to form the WOA-SA algorithm [22]. Hacer et al. combined the wrapper method and the filter method into an algorithm [23]. Zheng et al. proposed a new hybrid algorithm based on WOA and MPMD [24]. Carrasco et al. proposed a new filter wrapper feature selection method based on ranking [25]. Zhang et al. proposed a two-stage feature selection method for feature selection [26]. According to the combination method of wrapper and filter, the hybrid feature selection method is divided into two stages and embedded. In the two-stage method, the filter method is executed only once, and there is only one candidate feature subset provided for the wrapper method. Therefore, when searching for the optimal feature subset, the wrapper method has a limitation. In the embedded method, the close combination of the two methods increases the complexity of the algorithm and is easy to fall into the local optimum.

In order to eliminate the uniqueness of candidate feature subsets in the two-stage method, and to prevent the embedded method from falling into the local optimum, in this article, we employ the oscillation theory and combine the filter method and the wrapper method to propose a novel algorithm. This algorithm is called the Maximum Kendall coefficient Maximum Euclidean Distance Improved Gray Wolf Optimization algorithm (MKMDIGWO). In MDMKIGWO, Maximum Kendall coefficient Maximum Euclidean Distance (MDMK) algorithm is a filter feature selection method. The improved grey wolf optimization algorithm and the support vector machine (IGWO+SVM) are composed of a wrapped algorithm. In MDMKIGWO, after the adjustment of the oscillation theory, the MKMD algorithm provides multiple candidate feature subsets for the wrapper method, and the wrapper method searches for the optimal feature subset in multiple candidate feature subsets.

Based on the MRMR idea, MKMD filter algorithm is proposed, using Kendall to measure the correlation between features and labels, and using Euclidean distance to measure the compatibility between features. Two parameters (a and b) are introduced in MKMD to improve the correlation and reduce the redundancy of candidate feature subsets. The wrapper method uses an improved grey wolf optimization to improve the position update formula. By comparing the maximum number of retention times with the multiple values of the damping oscillation, the filter method and the wrapper method alternately perform depth and breadth search on the data set, and finally find the optimal feature subset. Therefore, the MKMDIGWO algorithm has higher classification accuracy on 12 data sets than the other 4 algorithms.

The rest of the paper is arranged in this way. The second part introduces the basic concepts of damping oscillation theory, gray wolf optimization and support vector machine. The third part shows the new algorithm and specific content. The fourth part describes the experimental results and experimental analysis. The fifth part draws the conclusions of this paper.

## Relation work

The most basic feature selection methods are the wrapper method and the filter method [27]. With the deepening of feature selection research, many researchers have merged the two basic methods to form a hybrid feature selection method. QASEM et al. proposed a binary version of the hybrid grey wolf optimization (GWO) and particle swarm optimization (PSO) [28]. RANYA et al. proposed a hybrid feature selection algorithm based on GWO and Harris Hawks Optimization (HHO) for feature selection [29]. Zheng et al. combined MSMC (Maximum Spearman Minimum Covariance) and CS (Cuckoo Search) algorithms to form MSMCCS algorithm [30].

In [28], to find the best solutions, the wrapper-based method K-nearest neighbors classifier with Euclidean separation matric is utilized. For performance evaluation of the proposed binary algorithm, 18 standard benchmark datasets from UCI repository are employed. The BGWOPSO algorithm is superior to the other four algorithms in accuracy, selecting the best optimal features, and the computational time.

In [29], a binary hybrid GWO and HHO was proposed. It can balance exploration and development through the combined binary GWO algorithm and HHO algorithm. In [30], a new filter algorithm (MSMC) was proposed. In hybrid algorithm, The MSMC algorithm is embedded in the CS algorithm. CS can select the optimal feature subset from the candidate feature subset provided by MSMC.

The above algorithm can show good performance on the specified data set, but it may decrease on other types of data sets. So an algorithm cannot solve the feature selection problem on all data sets. Therefore, the existing algorithms can be merged or improved to solve the feature selection problem. Next, we will discuss related theories and then clearly explain the proposed algorithm.

## The relation theory

In this part, we will introduce four important theories, namely damping oscillation theory, gray wolf optimization algorithm and support vector machine. These theories lay the foundation for the MKMDIGWO algorithm.

### Damping oscillation theory

Damping oscillation [31,32] refers to the vibration that the amplitude of the vibration system gradually attenuates with time due to friction and medium resistance or other energy consumption, also known as damping oscillation and damping oscillation. Whether it is a spring vibrator or a single pendulum, since external friction and medium resistance always exist, in the process of vibration, it must constantly overcome the external resistance to do work, consuming energy, and as the amplitude will gradually decrease, after a period of time, the vibration will completely stop. This vibration whose amplitude decreases with time is called damping oscillation. Because the amplitude is related to the energy of the vibration, the damping oscillation is also the vibration whose energy is decreasing. Damping oscillation is a non-harmonic motion. The damping oscillation system is a dissipative system. Damping here refers to the characteristics of any vibration system in the vibration, the amplitude of the vibration gradually decreases due to the external action or the inherent causes of the system itself, and the quantitative characterization of this characteristic.

In the ideal state, the spring device or the single pendulum device can complete the energy conversion, and the potential energy is converted into kinetic energy, thus the infinite conversion with no energy being lost, but in reality, whether it is the spring vibrator or the single pendulum due to the existence of external friction and medium resistance, in the process of vibration, the external resistance must constantly be overcome to do work and consume energy, and as the amplitude will gradually decrease, after a period of time, the vibration will stop completely. This is the cause of the damping oscillation.

**Fig 1:**
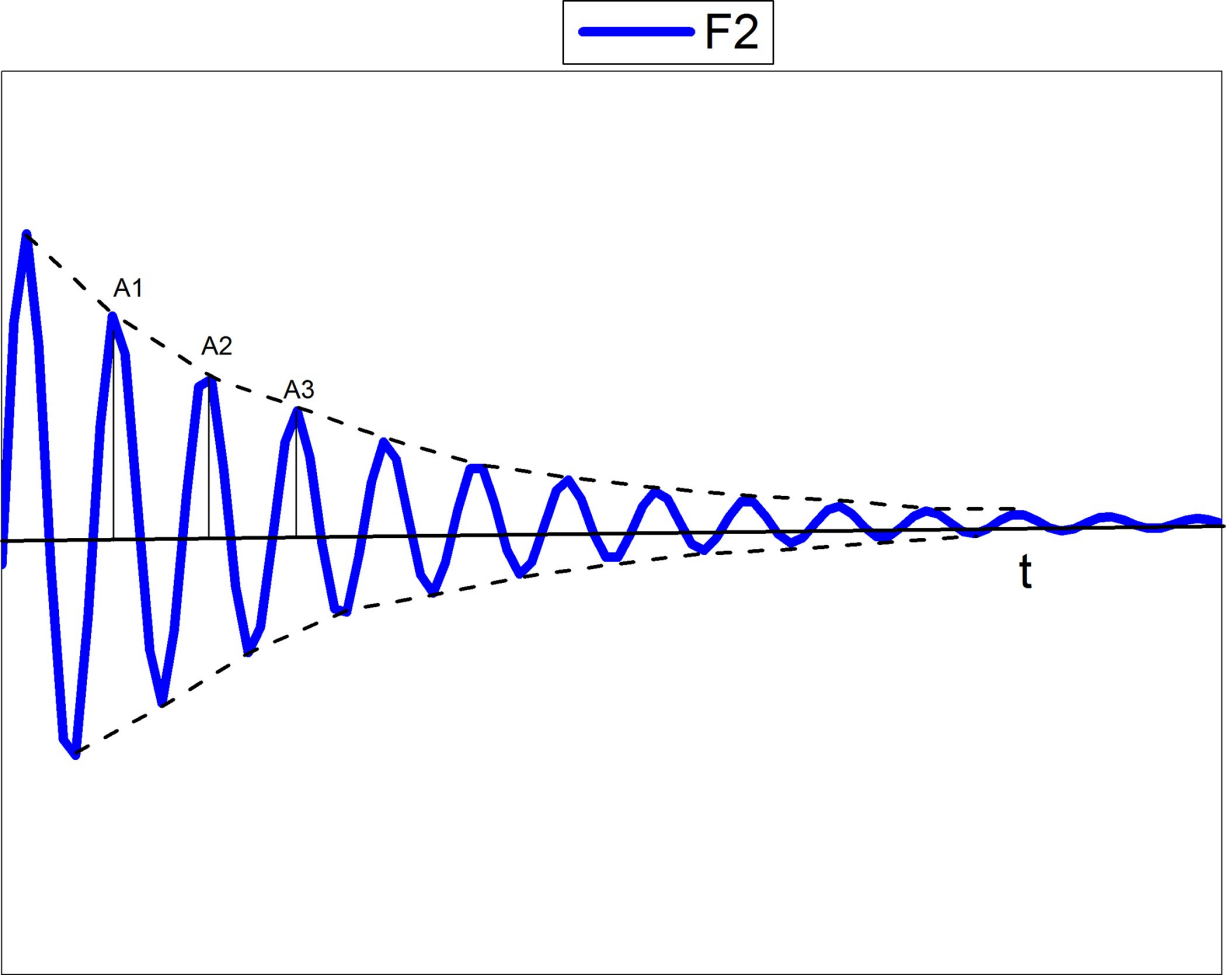
Damping oscillation curve.

The curve of the damping oscillation is shown in the Fig 1.

The curve in the figure can be expressed by the formula (1).


$${X=\mathrm{Ae}}^{‐\mathrm{nt}}\sin(\omega_{d}t+\varphi )$$ (1)

Where Ae-nt represents the envelope of the attenuation curve and t represents time.

According to the figure above, we can conclude that, the amplitude is gradually decreasing over time. It can be seen from the formula that the curve of the damping oscillation is composed of the product of the exponential function and the sine function.

### Gray wolf optimization

#### Continuous gray wolf optimization algorithm

**Fig 2:**
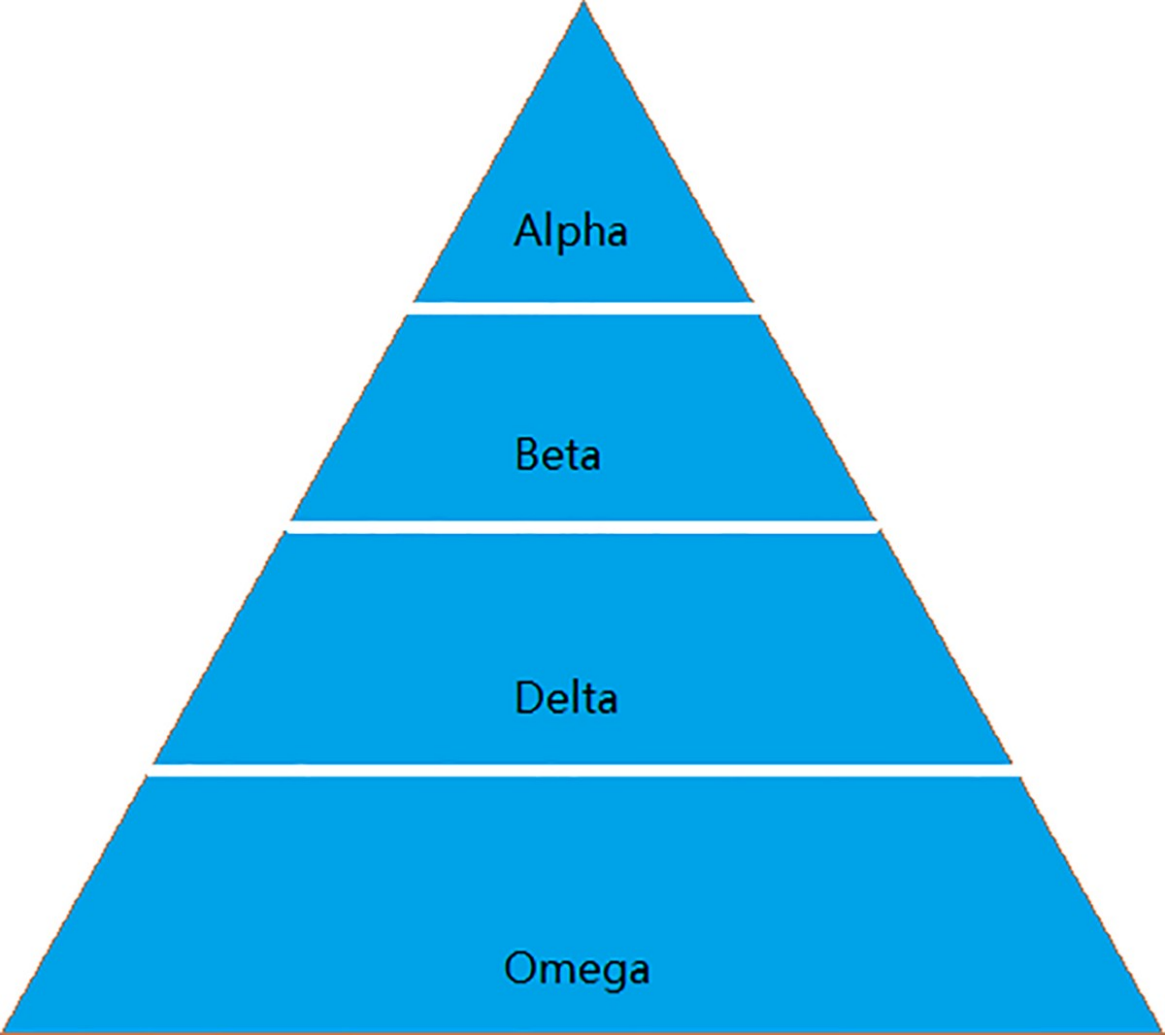
Gray wolf ranks.

In 2014, Mirjalili et al. imitated the living habits of the natural gray wolf and proposed the grey wolf optimization algorithm [33]. According to the characteristics of the gray wolves, they are divided into two parts. The first part is the leadership, and the second part is the general public. The leadership is arranged according to the level of the hierarchy as follows alpha (α), beta (β), gamma (δ). The general public layer consists of Omega (ω). According to the hunting habits of the gray wolf, the predation process of the wolves is divided into four steps. They are searching for prey, surrounding prey, attacking prey and preying on prey. According to the grouping and predation characteristics of wolves, in the constructed data model, alpha is considered the optimal solution, beta (β) is the second optimal solution, gamma (δ) is the third optimal solution, and Omega (ω) is the remaining candidate solution. The gray wolf grade model is shown in Fig 2.

According to the process of encircling prey during wolf hunting, the following formula is used to express its hunting behavior [33]:

$$\vec{D}=|\vec{C}*{\vec{X}}_{p}(t)‐\vec{X}(t)|$$ (2)

$$\vec{X}(t+1)={\vec{X}}_{p}(t)‐\vec{A}*\vec{D}$$ (3)

In formulas (2) and (3), t represents the t-th iteration, $\vec{x}(t)$ represents the position of the gray wolf in the t-th iteration, and $\vec{x}(t+1)$ represents the position of gray wolf in the (t+1)th iteration. ${\vec{x}}_{p}(t)$ indicates the position of the prey at the t-th iteration. $\vec{A}$ and $\vec{C}$ are vector coefficients, which are calculated by formulas (4) and (5), respectively [33].


$$\vec{A}=2*\vec{\alpha }*{\vec{r}}_{1}‐\vec{\alpha }$$ (4)

$$\vec{C}=2*{\vec{r}}_{2}$$ (5)

In these two formulas, r_1_ and r_2_ are random vectors whose range is [0, 1]. $\vec{a}$ is a linear decreasing function that varies with the number of iterations.

The update formula for the optimal solution (alpha) is calculated by formulas (6) and (7), and other optimal solutions (beta and delta) are similar. In the next iteration, the effects of the three optimal values are expressed by Eq (8).


$${\vec{D}}_{\alpha }=|{\vec{C}}_{1}*{\vec{X}}_{\alpha }‐\vec{X}|$$ (6)

$${\vec{X}}_{1}={\vec{X}}_{\alpha }‐{\vec{A}}_{1}*{\vec{D}}_{\alpha }$$ (7)

$$\vec{X}(t+1)=\frac{{\vec{X}}_{1}+{\vec{X}}_{2}+{\vec{X}}_{3}}{3}$$ (8)

In the continuous gray wolf optimization algorithm, the wolves start from the randomly assigned position information. After much iteration, under the leadership of the three wolves, the wolves move toward the optimal value and finally obtain the optimal value. In this iterative process, if $|\vec{A}|>1$ the candidate solution is far from the optimal value, if $|\vec{A}|<1$ the candidate solution is close to the optimal value. Finally, the GWO algorithm is terminated with the initially set criteria.

#### Discrete grey wolf optimization

Continuous gray Wolf optimization algorithm is not suitable for solving discrete problems. In 2016, Emary et al. proposed binary gray Wolf optimization algorithm to solve feature selection problem on the basis of continuous type [34]. In that paper, two discrete grey Wolf optimization algorithms, bGWO1 and bGWO2, are proposed, which are called the first discrete algorithm and the second discrete algorithm respectively in this paper.

In the first discrete algorithm, the position update strategy is calculated using formula (9) [34].


$$X_{d}{}^{t+1}=\{\begin{array}{l} X_{1}{}^{d}\;rand<\frac{1}{3}\\ X_{2}{}^{d}\;\frac{1}{3}\leq rand<\frac{2}{3}\\ X_{3}{}^{d}\;otherwise \end{array}$$ (9)

In this formula, X_1_^d^, X_2_^d^, X_3_^d^ represent the positions of alpha (α), beta (β), and gamma (δ) in the d dimension, respectively. X_d_(t+1) indicates the current wolf’s position in the d-dimension at (t+1) iterations, and rand is a random number whose range is [0,1]. The X_1_^d^ in the formula (9) is calculated by the formula (10), and the X_2_^d^, X_3_^d^ formula is similar to the X_1_^d^ formula.


$$X_{1}{}^{d}=\{\begin{array}{cc} 1 & (X_{\alpha }{}^{d}+bstep_{\alpha }{}^{d})\geq 1\\ 0 & otherwise \end{array}$$ (10)

In formula (10), X_α_^d^ represents the vector position of alpha (α) gray wolf in d dimensions. bstep_α_^d^ represents a binary value of alpha (α) gray Wolf in d dimension, which is calculated by formula (11).


$$bsetp_{\alpha }{}^{d}=\{\begin{array}{cc} 1 & cstep_{\alpha }{}^{d}\geq rand\\ 0 & otherwise \end{array}$$ (11)

In formula (11), rand has the same meaning as rand in formula (9). cstep_α_^d^ is a continuous value in d dimension. It is calculated by formula (12).


$$cstep_{\alpha }{}^{d}=\frac{1}{1{+e}^{{‐10*(A}_{1}{}^{d}{*D}_{\alpha }{}^{d}‐0.5)}}$$ (12)

In formula (12), A_1_^d^, D_α_^d^ are calculated by formula (2) and (4)

Based on the first discrete algorithm, the second discrete algorithm produces: transforming Eq (8) into Eq (13) [34].


$$X_{d}{}^{t+1}=\{\begin{array}{cc} 1 & sigmoid(\frac{x_{1}{}^{d}+x_{2}{}^{d}+x_{3}{}^{d}}{3})\geq rand\\ 0 & otherwise \end{array}$$ (13)

In formula (13), rand is a random number, X_d_^t+1^ represents the binary value of the d-dimensional position update in the t-th iteration, and sigmoid(a) is defined in formula (14) [34].


$$cstep_{\alpha }{}^{d}=\frac{1}{1{+e}^{{‐10*(A}_{1}{}^{d}{*D}_{\alpha }{}^{d}‐0.5)}}$$ (14)

The two discrete GWO algorithms proposed by Emary have more computational and understanding complexity. It is not conducive to the understanding and widespread use of researchers. In the third part, we will modify the discrete GWO algorithm to make the algorithm easier to understand and calculate.

### Support vector machines

Support Vector Machine (SVM) is a commonly used classifier. It is based on the SVM algorithm proposed by Vapnik in 1995 [35]. In both the two-category problem and the multi-classification problem, the SVM shows good performance [36].

In the LIBSVM classification process, there are two main steps to complete. The first step is to select the kernel function based on the data set. The second step is to train the kernel function using the data set. In the two steps, the most important is the first step. When selecting a kernel function, there are four kernel functions commonly used, and the formulas are given below.

The first is a linear kernel function, expressed by formula (15).


$$K(x_{i},x_{j})=x_{i}{}^{T}x_{j}$$ (15)

The second is a polynomial kernel function, expressed by formula (16).


$$K(x_{i},x_{j})={\left(\gamma x_{i}{}^{T}x_{j}+r\right)}^{d},\;\gamma >0$$ (16)

The third is the RBF kernel function, which is represented by formula (17).


$$K(x_{i},x_{j})=\exp(‐\gamma {\left|x_{i}‐x_{j}\right|}^{2}),\gamma >0$$ (17)

The fourth is the sigmoid kernel function, represented by formula (18).


$$K(x_{i},x_{j})=\tanh(\gamma x_{i}{}^{T}x_{j}+r)$$ (18)

Here r and d are nuclear parameters.

## MKMDIGWO algorithm

In this part, we propose the MKMDIGWO algorithm. Firstly, the MKMD filtering algorithm is proposed. Secondly, an improved discrete gray wolf optimization algorithm is proposed. Finally, the proposed MKMDIGWO algorithm is described.

### MKMD filter algorithm

In hybrid feature selection algorithms, filter algorithms are often used to rank features in a data set. Commonly used filter algorithms are IG, F-score, MRMR, MCMD, etc., which are either univariate or multivariate. The univariate filter algorithm considers more of the correlation between labels and features, while the multivariate filter algorithm increases the measure of redundancy between features. The well-known multivariate filter algorithm MRMR uses mutual information to measure the correlation between labels and features and the redundancy between features, the proportion of the two parts is the same. In this paper, a new filter algorithm MKMD is proposed. The Kendall Rank correlation coefficient is used to measure the correlation between labels and features, and the Euclidean distance is used to measure the redundancy between features. Then the two parameters (a and b) dynamically adjust the ratio of correlation coefficient and redundancy.

#### Maximum Kendall rank

In statistical theory, the Kendall rank factor is one of the methods widely adopted to measure the correlation between two vectors. The Kendall correlation coefficient is named after Maurice Kendall and often represented by the Greek letter τ (tau). The Kendall correlation coefficient ranges from -1 to 1. When τ is equal to 1, it means that two random variables have a consistent rank correlation; when τ is -1, it means that two random variables have the exact opposite rank correlation; when τ is equal to 0, it means that the two random variables are independent of each other.

To calculate the Kendall correlation coefficient, there exist three ways as introduced respectively below.

The first calculation method is expressed by the formula (19). This method is applicable to the case where the same elements do not exist in the set X and Y, that is, each element in the X set is unique (the Y set is the same).


$$T_{1}=\frac{2(C-D)}{N(N‐1)}$$ (19)

Where C is the pairs of the element with consistency in XY (two elements are a pair); D is the pairs of the element with inconsistency in XY. N represents the number of elements contained in X or Y.

The second calculation method is expressed by the formula (20). This method is suitable for the case where the same element exists in the vector X or Y.


$$T_{2}=\frac{C‐D}{\sqrt{{(N}_{3}{‐N}_{1}{)(N}_{3}{‐N}_{2})}}$$ (20)

In the formula (20), C and D are the same as in the formula (19). N3 is calculated by formula (21). N1 and N2 are obtained for the data characteristics of the set X and Y, respectively, and the specific values are calculated by the formula (22) and the formula (23).


$$N_{3}=\frac{1}{2}N(N‐1)$$ (21)

$$N_{1}=\sum_{i=1}^{s}\frac{1}{2}U_{i}(U_{i}-1)$$ (22)

$$N_{2}=\sum_{i=1}^{t}\frac{1}{2}V_{i}(V_{i}-1)$$ (23)

The calculation method of N1 is as follows. The same elements in X are respectively combined into a small set, and the number of these small sets in X is represented by s, and Ui represents the number of elements included in the i-th small set. N2 is calculated on the basis of the set Y. The same elements in Y are respectively combined into small sets, the number of these small sets in Y is represented by t, and Vi represents the number of elements included in the i-th small set.

The third calculation method is expressed by the formula (24).


$$T_{3}=\frac{2M(C‐D)}{N^{2}(M-1)}$$ (24)

In the formula (24), C, D, and N are the same as in the formula (19), and M represents the smaller one of the number of rows and the number of columns in the rectangular table. Eq (24) only applies to the calculation of the correlation coefficient between the random variables X and Y represented by the table, without considering the influence of the presence of the same element in the set X or Y on the final statistical value.

Assume that vectors X(4,1,1,1,2,3,3,4,5,6,7,3,4,1,2,2,2,2,3,3,5,5,5)’ and *Y*(4,4,0,4,4,4,4,0,0,4,4,2,0,0,4,6,2,4,0,0,2,2,4)’ are given. We can see that there are 7 different elements in vector X, of which 1–5 are repeated, and 6 and 7 only appear once. According to formula (22), s = 5, U1 = 4, U2 = 5, U3 = 5, U4 = 3, U5 = 4. From this, N1 = 35 is calculated. The same method according to formula (23), we can see that in vector Y, t = 3, V1 = 7, V2 = 4, V3 = 11, N2 = 82.

There are two random variables X and Y (also can be regarded as two sets), and their number of elements is N. The i-th (1< = i< = N) values of two random variables is represented by Xi, Yi respectively. The same position corresponding elements in X and Y constitute an element pair set KR, which contains elements (Xi, Yi) (1 < = i < = N). When any two elements (Xi, Yi) in the set KR have the same trend as (Xj,Yj), the two elements are considered to be consistent. In other words, when Xi>Xj, Yi>Yj is also true; or Xi<Xj and Yi<Yj are established at the same time. Conversely, when any two elements (Xi, Yi) and (Xj,Yj) in the set KR have different trends, the two elements are considered to be inconsistent. In other words, when Xi>Xj, Yi<Yj also holds; or Xi<Xj and Yi>Yj hold together.

Since the range of Kendall coefficient values is [–1, 1], we only consider integers when measuring random variables. Therefore, we use Eq (25) to calculate Kendall coefficients.


$$T(L,F)=\frac{\left|C‐D\right|}{\sqrt{{(N}_{3}{‐N}_{1}{)(N}_{3}{‐N}_{2})}}$$ (25)

**Fig 3:**
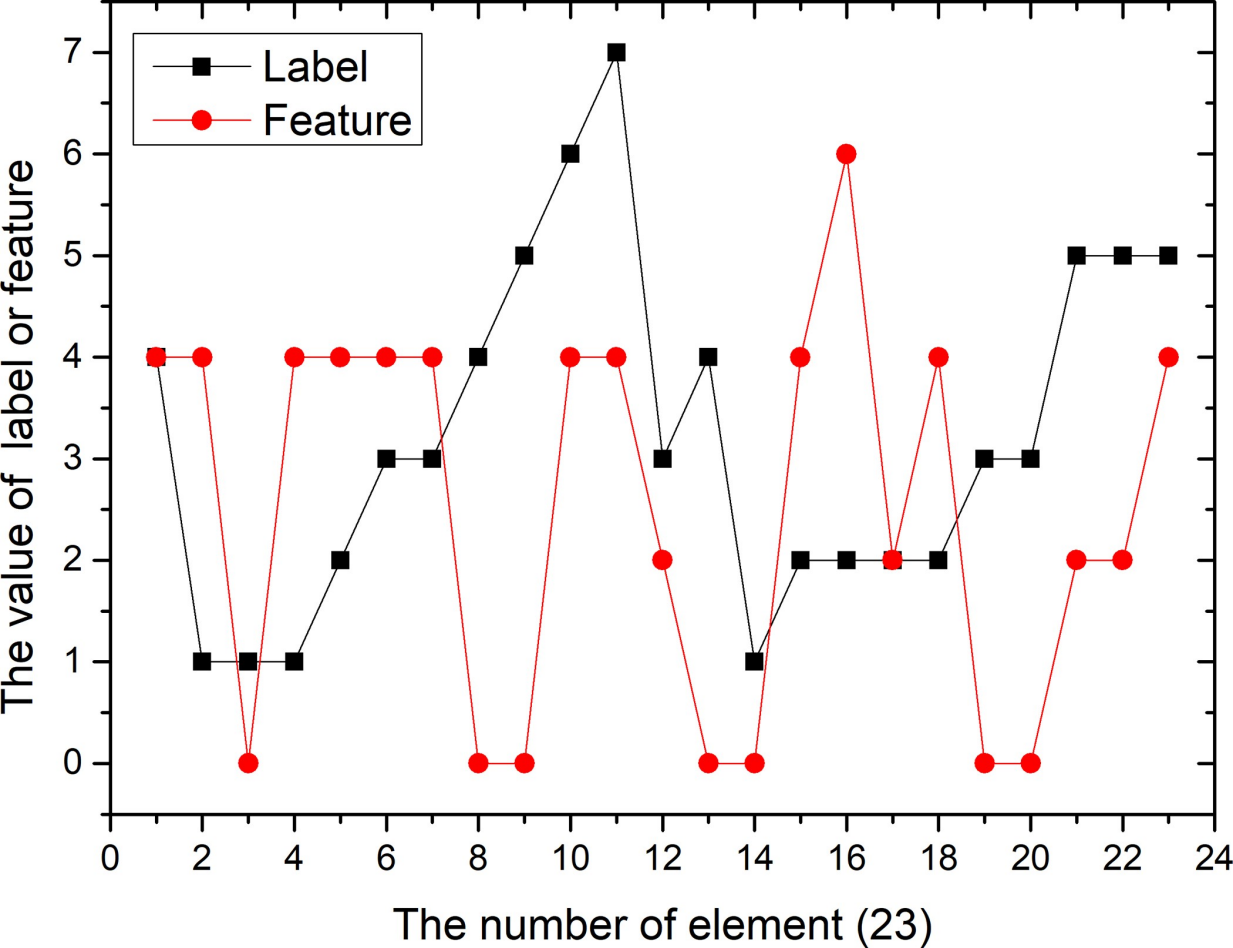
Trend about label (X) and feature (Y) (23 elements).

**Fig 4:**
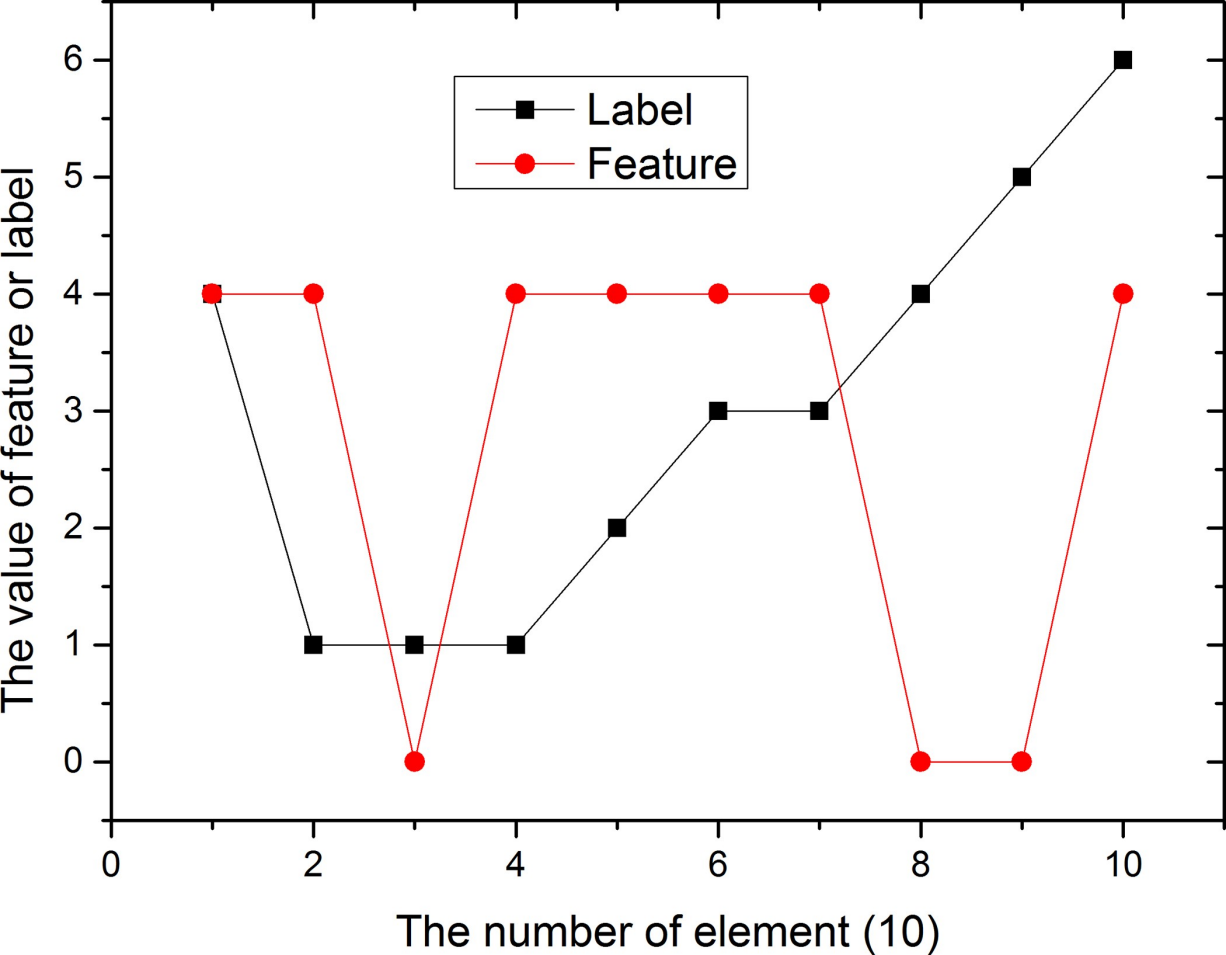
Trends in label X and feature Y (top 10 elements).

We consider the X vector as a label and the Y vector as a feature. The trends of the two are shown in Figs 3 and 4.

We use Eq (25) to calculate the Kendall coefficient for a given vector X and Y to be 0.046. It can be seen from the figure that the trends of the two are different and the correlation between the two is very low.

**Fig 5:**
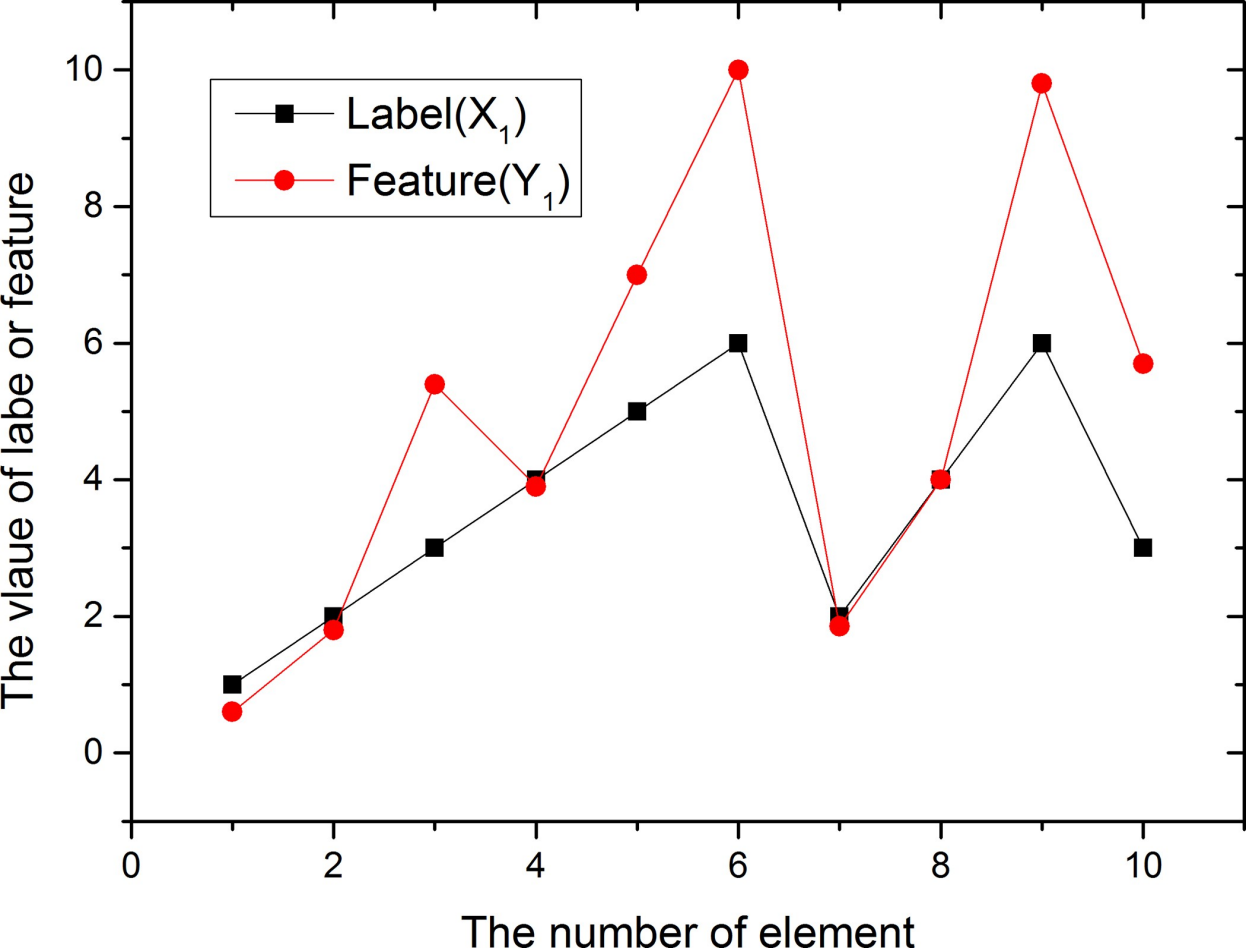
Trends in label X1 and feature Y1.

Assume that the vector *X*_1_(1,2,3,4,5,6,2,4,6,3)’ and *Y*_1_(0.6,1.8,5.4,3.9,7,10,1.85,4,9.8,5.7), the trend of the two are shown in Fig 5. Both Kendall coefficients are 0.7683.

The tree figures tell us that the correlation between X and Y is relatively small, and the correlation between X1 and Y1 is relatively large.

There are multiple features in the data set, and the feature Fi with the highest correlation with the tag is calculated by the formula (26).


$${\mathrm{MK}(F}_{i}{,L)=\max(T(L,F}_{i}))$$ (26)

Where Fi represents the i-th feature in the data set, L represents the tag vector in the data set, and max represents the most-valued function, i = 1, 2, 3…, n.

#### Maximum European distance

According to the idea of Maximum Relevance Minimum Redundancy, it is necessary to consider not only the correlation between labels and features but also the compatibility between features. Among the commonly used measures of compatibility between features are mutual information, Euclidean distance, cosine angle, and so on. The Euclidean Distance is adopted in this paper to measure the redundancy between features.

Euclidean distance is the easiest to understand distance calculation method, derived from the distance formula between two points in Euclidean space.

The Euclidean distance between two points a(x_1_, y_1_) and b(x_2_, y_2_) on a two-dimensional plane is expressed by formula (27).


$$D_{\mathrm{ab}}=\sqrt{{\left(x_{1}‐x_{2}\right)}^{2}{+(y}_{1}{‐y}_{2}{)}^{2}}$$ (27)

The Euclidean distance between two points a(x_1_, y_1_, z_1_) and b(x_2_, y_2_, z_2_) in three-dimensional space is expressed by the formula (28).


$$D_{\mathrm{ab}}=\sqrt{{\left(x_{1}‐x_{2}\right)}^{2}{+(y}_{1}{‐y}_{2}{)}^{2}{+(z}_{1}{‐z}_{2}{)}^{2}}$$ (28)

The Euclidean distance between two n-dimensional vectors a(x_11_,x_12_,…,x_1n_) and b(x_21_,x_22_,…,x_2n_) is expressed by formula (29).


$$D_{\mathrm{ab}}=\sqrt{\sum_{k=1}^{n}{\left(x_{1k}‐x_{2k}\right)}^{2}}$$ (29)

Two features are given as F_i_ and F_j_, respectively, and the Euclidean distance is denoted as D (F_i_, F_j_). It is expressed by the formula (30).


$${D(F}_{i}{,F}_{j})=\sqrt{{\left(F_{i1}{‐F}_{j1}\right)}^{2}+{\left(F_{i2}{‐F}_{j2}\right)}^{2}+\ldots +{\left(F_{\mathrm{in}}{‐F}_{\mathrm{jn}}\right)}^{2}}=\sqrt{\sum_{k=1}^{n}{\left(F_{ik}‐F_{jk}\right)}^{2}}$$ (30)

The value of the Euclidean distance represents the redundancy between the two features. The larger the distance value, the more the redundancy of the feature.

Give two features F_1_(1,5,1,1,1,1,1,2,2,1,1,1,1,1,1,1,1,1,1,1,1,1,2) and F_2_(1,4,2,2,1,2,2,2,1,1,2,1,2,2,2,1,2,1,1,2,1,1,1).The correlation distance D (F_1_, F_2_) = 1.1149 after data centering indicates that the redundancy is high.

The distance between the two features is expressed by Eq (30). According to a known feature, the feature of finding the feature Euclidean distance maximum in a feature set is represented by Eq (31).


$${\mathrm{MD}1(F}_{k}{,F}_{\mathrm{set}}{)=\max(D(F}_{k}{,F}_{j}))$$ (31)

Where F_k_ represents a known feature, F_Set_ represents a feature set, max function represents a maximum value, F_j_ is a feature in F_Set_. The distance between F_j_ and F_k_ is greater than the distance between other features and F_k_ features. D(F_k_, F_j_) is calculated using Eq (30).

A collection of selected features is called a selected feature set. A set of candidate features is called a candidate feature subset. A feature is selected from the candidate features subset, and the distance between the feature and the selected feature subset is required to be a maximum. The distance between the two feature sets is represented by Eq (32).


$${\mathrm{MD}2(F}_{\mathrm{setk}}{,F}_{\mathrm{set}}{)=\max(\mathrm{MD}1(F}_{m}{,F}_{\mathrm{setk}}))$$ (32)

Where F_Setk_ represents the selected feature set. F_set_ represents the candidate feature set. F_m_ represents the feature with the largest distance selected from the F_set_ set. MD1 (F_m_,F_Setk_) is calculated using Eq (31).

#### Maximum Kendall Maximum Distance (MKMD)

The maximum Kendall (MK) method measures the correlation between labels and features, and the Maximum Euclidean distance (MD) measures the redundancy between features. There are two ways to combine MK and MD into a formula, one method is MK multiplied by MD, the other method is MK added by MD. To reduce the amount of calculation, here we choose to add. Combining the contents of the first two sections, the MKMD method of selecting features (F_j_) one by one from the subset of candidate features is represented by formula (33).


$${\mathrm{MKMD}(F}_{j})=\{\begin{array}{ll} \;\mathrm{MK}(F_{j},L) & i=0,j=1\\ \;\mathrm{MK}(F_{j},L)+\mathrm{MD}1(F_{j},F_{\mathrm{Set}}) & i=1,j=2\\ {\mathrm{MK}(F}_{j}{,L)+\mathrm{MD}2(F}_{\mathrm{Setk}}{,F}_{\mathrm{set}}) & i>1,j>2 \end{array}$$ (33)

Where i represent the number of features that have been selected before executing formula (33), and F_j_ represent the selected features using this formula.

In (33), the roles of MK and MD are always the same. From the process of selecting features, when the number of features is gradually increased, the redundancy between features is greater than the correlation between features and labels. Therefore, two parameters (a and b) are added to the Eq (33) to adjust the role of correlation and redundancy.


$${\mathrm{MKMD}(F}_{j})=\{\begin{array}{ll} \;a*\mathrm{MK}(F_{j},L) & i=0,j=1\\ \;a*\mathrm{MK}(F_{j},L)+b*\mathrm{MD}1(F_{j},F_{\mathrm{Set}}) & i=1,j=2\\ {a*\mathrm{MK}(F}_{j}{,L)+b*\mathrm{MD}2(F}_{\mathrm{Setk}}{,F}_{\mathrm{set}}) & i>1,j>2 \end{array}$$ (34)

Where a and b are parameters used to adjust the effects of both the maximum correlation and the maximum distance. Since the values of MK and MD on different data sets are quite different, the values of a and b cannot be 1, and the value of a changes from large to small, and the value of b increases from small to large. Formula (35) and (36) give values for a and b.


$$a=1-{\left(\frac{t}{T}\right)}^{2}$$ (35)

$$b={\left(\frac{t}{T}\right)}^{2}$$ (36)

**Fig 6:**
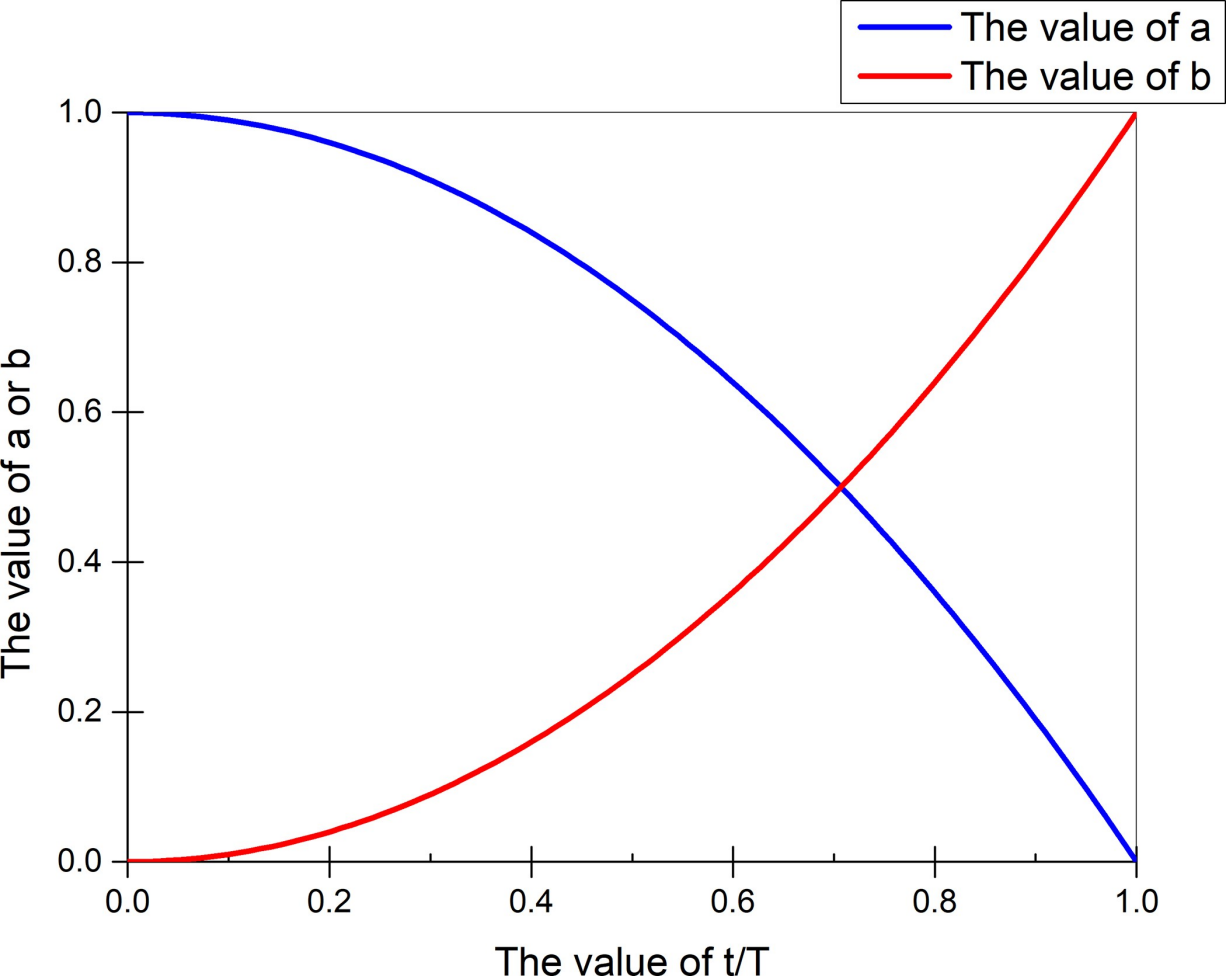
a and b change trend chart.

Where t represents the current number of iterations and T represents the total number of iterations. The trends of a and b can be seen from Fig 6. a gradually decreases from the maximum value of 1 to 0. b gradually increases from a minimum value of 0 to 1. a is gradually decreasing, and b is gradually increasing. Both are equal values at t = 70. According to the weights represented by a and b, it can be seen from the curve that the correlation effect of MK is greater than MD when t<70, and the effect of MD is greater when t>70.

**Fig 7:**
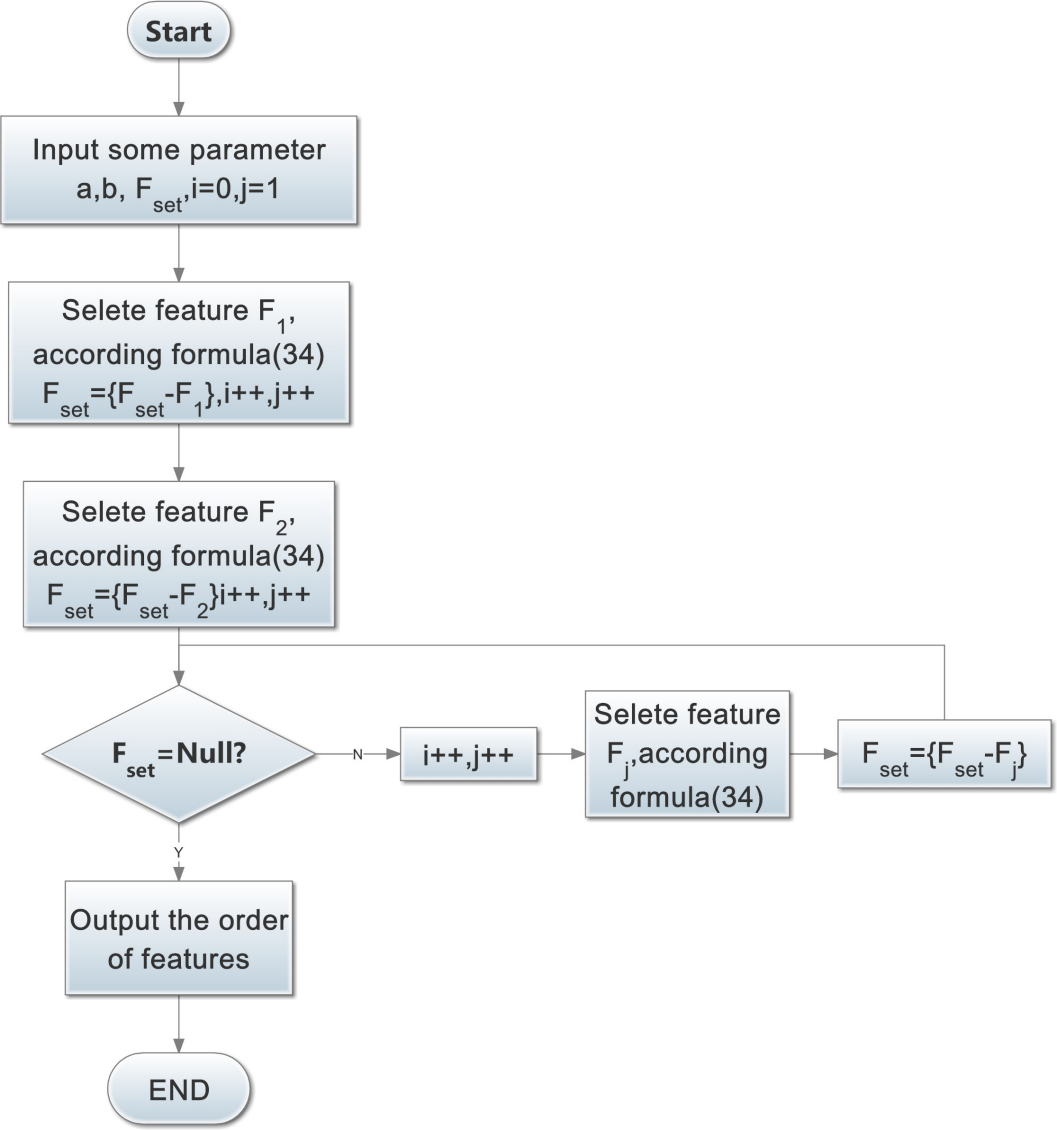
MKMD algorithm flow chart.

The pseudo code of the MKMD algorithm is shown in Algorithm 1, and the flow chart is shown in Fig 7.

Algorithm 1. MKMD algorithm pseudo code.

1 **Input** Parameter: the number of feature: F_set_,a,b,i = 0,j = 1

2 **Output**: the order of feature

3 Select feature F_1_ from Fset according to formula (34) (i = 0)

4 F_set_ = F_set_-{F_1_} i = i+1,j = j+1

5 Select feature F_2_ from Fset according to formula (34) (i = 1)

6 F_set_ = F_set_ -{F_2_}, i = i+1,j = j+1;

7 **While** Fset<>Null

8 Select feature F_j_ from Fset according to formula (34) (i>1)

9 F_set_ = F_set_-{F_j_}

10 j = j+1

11 i = i+1

12 **Wend**

### Improved grey wolf optimization

In two typical discrete GWO algorithms, updating the positional formulas of the three leaders requires multiple formulas. This paper is an improvement on the gray wolf algorithm. To calculate each position of each gray wolf, what should be considered includes the three most important values for the current impact, as well as the position in the previous iteration. Therefore, the value of the current position is generated by two parts, which are, respectively, the three most value and the value in previous iteration. These two parts are represented by thrg(x_1_, x_2_, x_3_) and xpre(i, j), respectively. x_1_, x_2_, x_3_ respectively represent the position information of the three wolves in the wolf group in the discrete gray wolf algorithm, which is calculated by formula (9). xpre(i,j) represents the position information of the current position in the latest iteration. Therefore, the value of xpre(i,j) does not need to be given a calculation formula. The value of Thrg(x_1_, x_2_, x_3_) is determined by formula (41). The update formula of the current position X(i,j) can be found in the formula (37).


$$x(i,j)=t_{1}*thrg(x_{1},x_{2},x_{3})+t_{2}*xpre(i,j)$$ (37)

Among them, t_1_ and t_2_ are parameters, which are used to mediate the different influence weights of the two parts on the current position. For the calculation methods of t_1_ and t_2_, see Eqs (38) and (39).


$$t_{1}=\sin(x)*t\;0<x<\frac{p}{2}$$ (38)

$$t_{2}=1‐4*t*\sin(x)\;0<x<\frac{\pi }{2}$$ (39)

Where x is arbitrarily taking a number within the specified range, t is a linear decreasing function. Formula (40) is used to calculate t.


$$t=\frac{T-l}{T}$$ (40)

Where l represents the current number of iterations and T represents the maximum number of iterations.

The first discrete algorithm uses the segmentation function to select only one of them as a representative of three at a time, and the other two max values have no effect. The second discrete algorithm considers all three effects, but the calculation is more complicated. The method proposed in this paper considers the effects of three influencing results, while the amount of calculation is small. The thrg (x_1_, x_2_, x_3_) is calculated by formula (41).


$${\mathrm{thrg}(x}_{1}{,x}_{2}{,x}_{3})=\{\begin{array}{ccc} 1 & \left|x_{1}{+x}_{2}{+x}_{3}|>3\sin(x\right) & x=\mathrm{rand}*\frac{\pi }{2}\\ 0 & \mathrm{otherwise} \end{array}$$ (41)

x_1_, x_2_, x_3_ represent the position information of the three-headed wolf, and the range is [–1, 1]. The absolute value of the sum of the three is in the range of (0, 3). The value range of 3sin(x) is in the range of (0, 3). Rand is a random number. In the improved gray wolf algorithm, under the guidance of the three-headed wolf, the optimal feature subset can be found through iteration.

### MKMDIGWO algorithm

#### Damping oscillation adjustment

The filter algorithms and optimization algorithms mentioned in A and B can be combined in various ways, such as wrapper, filter, and hybrid. Among the various methods, the hybrid method is the most commonly used. This paper introduces the principle of damping oscillation and dynamically adjusts the combination of filter algorithm and wrapper algorithm. The wrapper algorithm is used to perform multiple optimizations based on the different results of the filter algorithm.

According to formula (1), a new damping oscillation formula (42) is proposed.


$$y=\lceil \left|{2*(e}^{\left(2‐\frac{t}{60}\right)}*\cos(\frac{t}{6}))\right|\rceil +\lceil 5*\mathrm{rand}\rceil$$ (42)

In the formula, t represents the number of iterations, which varies from 0 to 100, and y represents the amplitude value at each time t. In the original damping oscillation, the product of the exponential function and the sine function is used. The proposed function replaces the sine function with a cosine function because the algorithm does not undergo any iterations for optimization at time t. Since the cosine function has a negative number, which is meaningless to us, an absolute value is taken for the entire result. Rand represents a random number ranging from 0 to 1, and this symbol ⌈ ⌉ indicates rounding up. Increasing the random number means that the value of y is not fixed at each execution.

**Fig 8:**
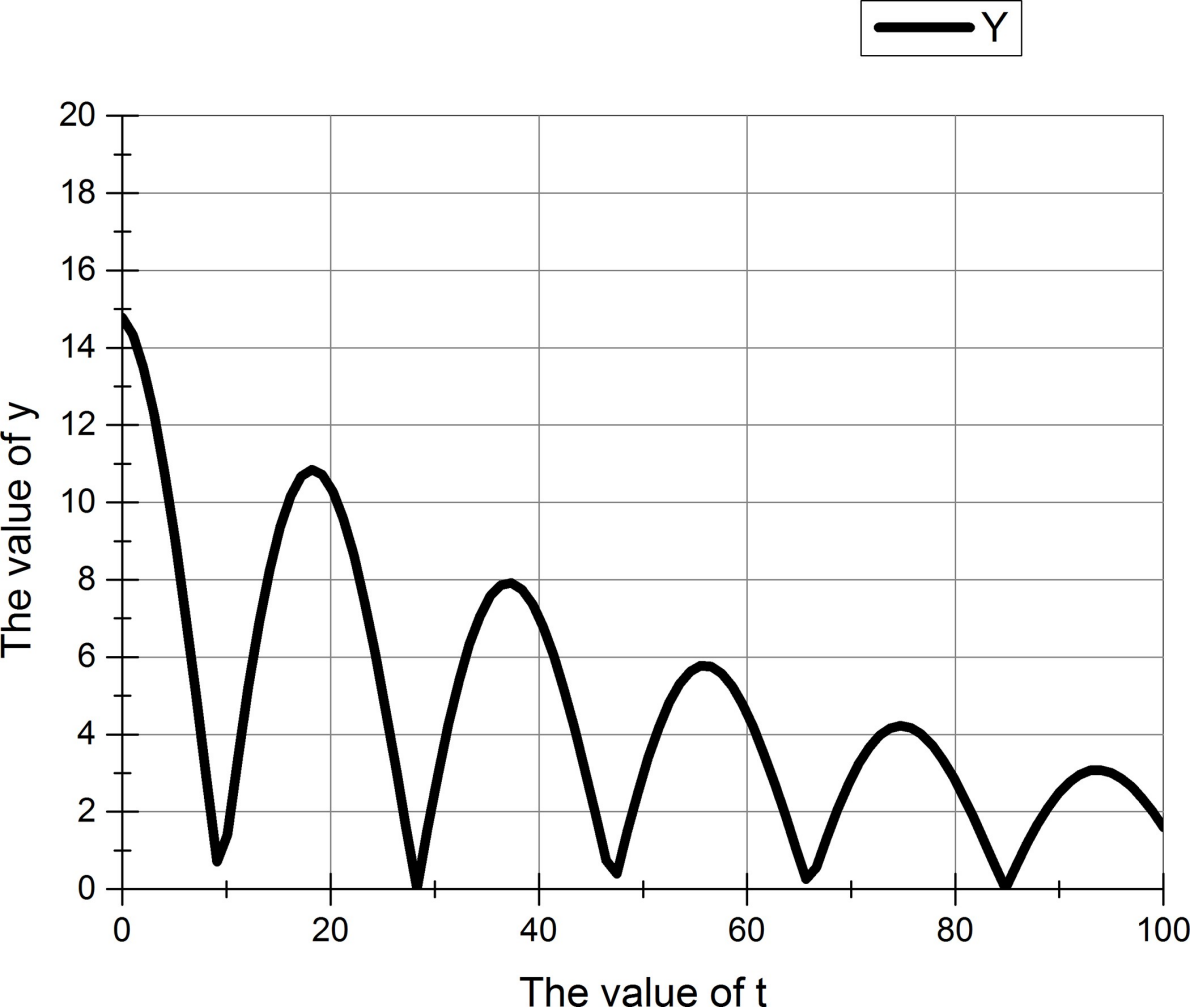
Damping oscillation function (before adding random numbers).

**Fig 9:**
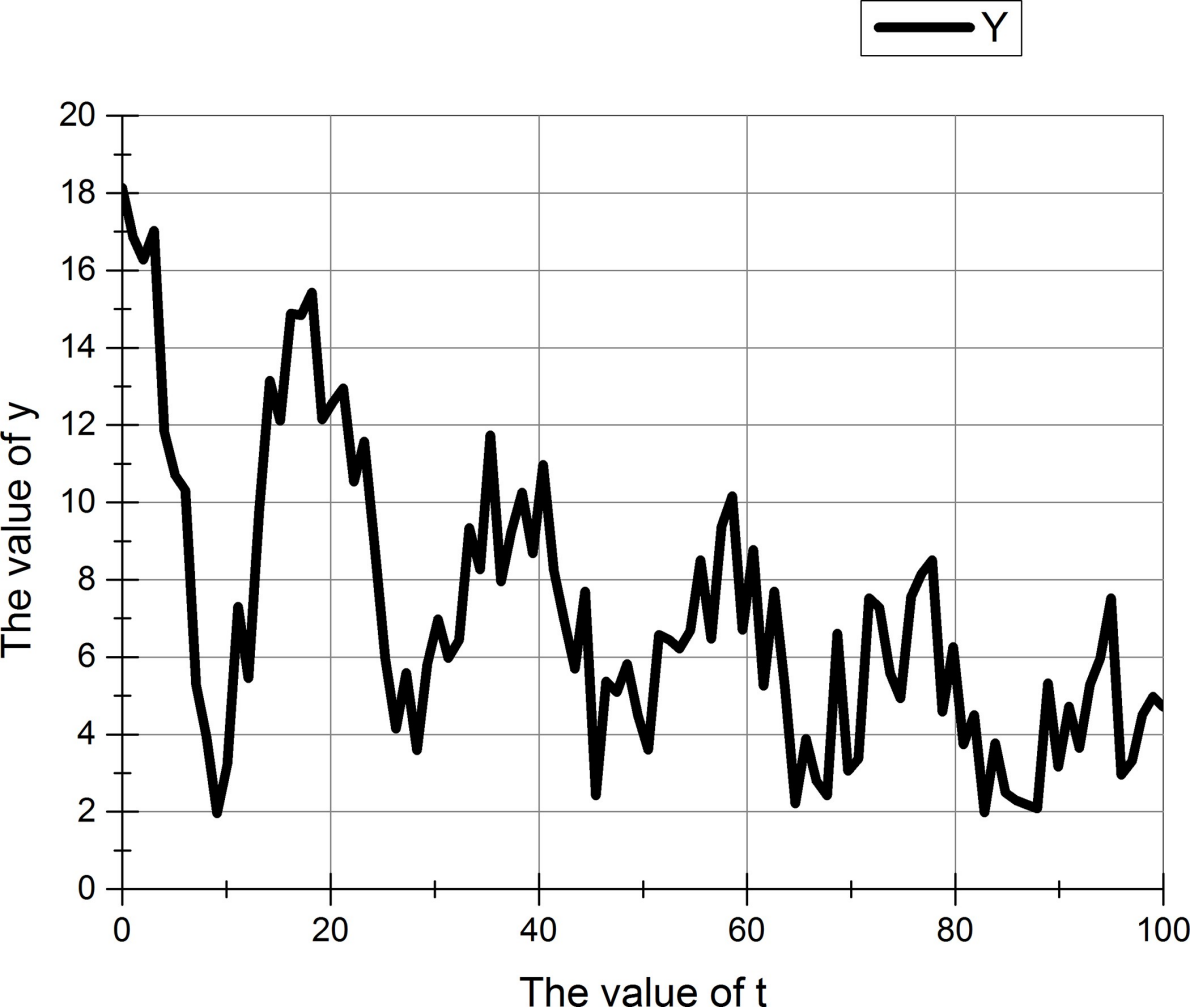
Damping oscillation function (after adding random numbers).

The two figures (Figs 8 and 9) show that whether or not to increase the random number, y has six maximum values during the change process. These six maximum values are used to adjust the execution of the wrapper algorithm and the filter algorithm.

#### MKMDIGWO algorithm framework

**Fig 10:**
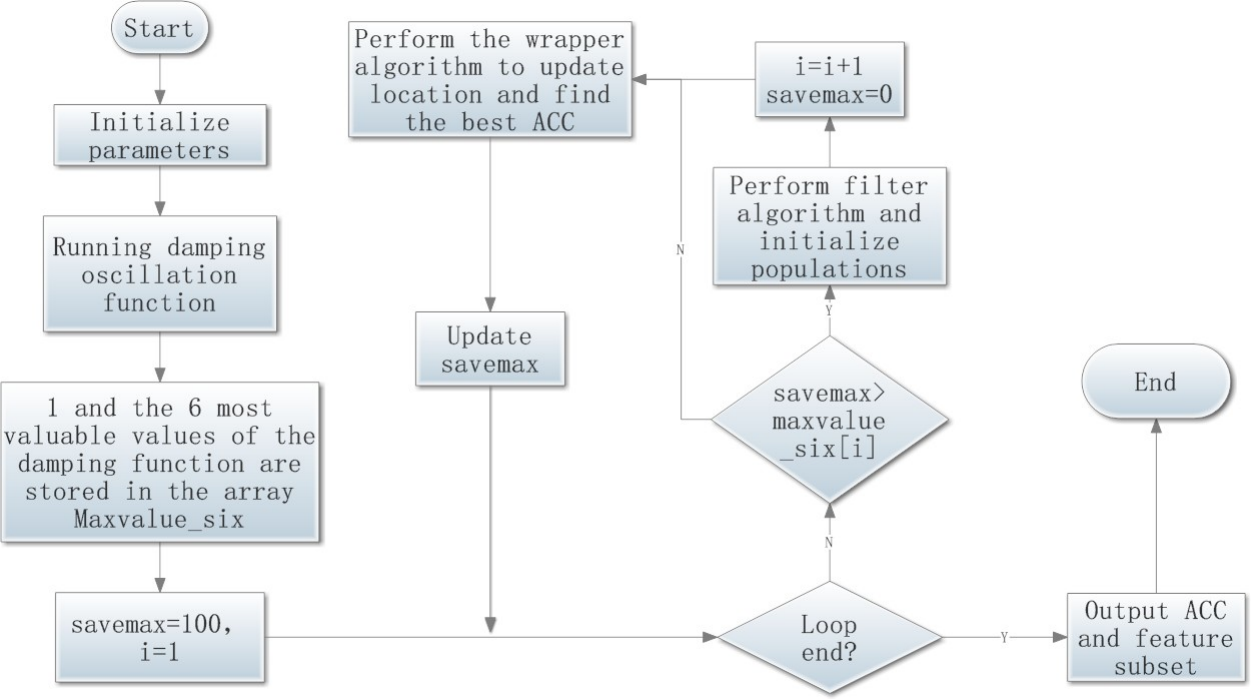
MKMDIGWO algorithm framework.

The framework of the MKMDIGWO algorithm is shown in the Fig 10. First the parameters are initialized and then the damping oscillator function is executed. The six max values (maxvalue_six) of the damping oscillator function adjust the execution of the filter algorithm and the optimization algorithm. An initial value of the maximum number of reservations (savemax) is assigned to 100.

After these preparations being completed, the program enters the loop section, in which it is determined whether to execute the filter algorithm according to the relationship between the maximum value retention number (savemax) and the damping oscillation maximum value (maxvalue_six). Due to the initial value setting, in the first loop, the filter algorithm is executed and the population is initialized, then the optimization algorithm is executed to realize the population position update and find the optimal value. Then the value of savemax is updated.

In the other cycles after the first cycle, the relationship between the savemax and the maxvalue_six is first determined.

If the value of savemax is bigger than the maxvalue_six, it is indicated that the obtained maximum value has not been updated many iterations. The optimal value found on this basis is already the maximum value, and there is no need to recycle. It is necessary to change to an initial state to continue searching for optimization. Therefore, the filter algorithm is re-executed. The population is reinitialized. To recalculate, the value of savemax is set to 0.

If the value of savemax is smaller than the maxvalue_six, it is indicated that the optimization can be continued on this basis, and the filter algorithm does not need to be re-executed. Therefore, the maximum value of the damping oscillation (maxvalue_six) determines the execution of the filter algorithm.

In one certain cycle, the highest value of the classification accuracy after optimization by the optimization algorithm is compared with the maximum value in the previous cycle. If the former is greater than the latter, the maximum value retention number (savemax) is assigned 1; otherwise, the value of savemax is automatically incremented by 1. Under the multiple adjustment of the damping oscillation, the maximum value of classification accuracy is obtained in many classification accuracies, and the maximum value is selected as the output information.

## Experimental results and analysis

In this part, we will introduce experimental results and analysis. First, the data set used in the experiment is described. Second, we introduce the settings of the parameters used by each algorithm in the experiment. Finally, the results and analysis of the experiment are introduced.

### Experimental data

Experimental data are available on UCI Machine Learning and the website (http://www.gems-system.org/, Gene Expression Model Selector). Based on these data sets, the proposed algorithm is evaluated. Table 1 describes the details of these data sets. There are a variety of classified data sets, including 2 categories, 3 categories, 5 categories, and 11 categories. The number of instances in the dataset is between 102 and 1000. The number of characteristics of the data set is between 13 and 1993. From the above information, it can be concluded that these data sets contain a wide range.

**Table 1:** Description of the data set used in the experiment.

NO.	Dataset	# of instances	#of features	# of classes	abbreviation
1	11_Tumors	174	12534	11	11t
2	Breast-Cancer-Wisconsin-Diagnostic	569	30	2	BD
3	Breast-Cancer-Wisconsin-Prognostic	198	34	2	BP
4	Detect	373	531	2	DE
5	German	1000	25	2	GE
6	Lung_Cancer	203	12601	5	LU
7	SPECT-Heart-SPECTF	267	45	2	SH
8	Echocardiogram	133	13	2	EC
9	Parkinsons	195	23	2	PA
10	Prostate_Tumor	102	10509	2	PT
11	SMK-CAN-187	187	19993	2	SC
12	wine	178	14	3	WI

### Experimental parameter setting

In the MKMDIGWO algorithm, a filter algorithm, MKMD, and a wrapper algorithm are included. In the wrapper algorithm, the number of iterations is 100 and the number of wolves is 30. Though there are some papers on wrapper algorithms and filter algorithms, it is difficult to find other algorithms that are exactly the same as the MKMDIGWO algorithm. For a fair comparison, we reproduce some algorithms, such as MRMR+ PSO, MRMR+GA, MRMR+BBA, MRMR+CS. In the four algorithms, the filter method also uses the mid method of the MRMR algorithm. The number of iterations and the number of populations are the same as MKMDIGWO. The initialization parameters and implementation methods of the four algorithms are referred to reference [37–41]. The parameter settings of the four algorithms are shown in Tables 2–5.

**Table 2:** Detailed parameter values of the mRMR+PSO algorithm.

Parameter	Description	Value
population	Number of particles	30
Wmax	Maximum value of weight	0.9
Wmin	Minimum value of weight	0.4
C1,C2	Coefficients	2
Iteration max	Maximum number of iterations	100
K	Number of selected genes for mrmr	100

**Table 3:** Detailed parameter values of the mRMR+GA algorithm.

Parameter	Description	Value
N	The number of chromosomes	30
Pc	Crossover probability	0.7
Pm	Mutation probability	0.02
Iteration max	Maximum number of iterations	100
K	Number of selected genes for mrmr	100

**Table 4:** Detailed parameter values of the mRMR+BBA algorithm.

Parameter	Description	Value
N	The number of bats	30
L	Loudness	1.5
P	Pulse rate	0.5
Qmin	Minimum value of frequency	0
Qmax	Maximum value of frequency	1
Iteration max	Maximum number of iterations	100
K	Number of selected genes for mrmr	100

**Table 5:** Detailed parameter values of the mRMR+CS algorithm.

Parameter	Description	Value
N	The number of bats	30
R	Discovery rate of alien eggs/solution	0.25
C	The parameter of Levy flight	1.5
Iteration max	Maximum number of iterations	100
K	Number of selected genes for mrmr	100

To make the results of the experiment more stable, each algorithm was executed 10 times on each data set. The classification accuracy rate of each data set has 2 values, which are the maximum of 10 times and the average of 10 times. In this study, the SVM classifier is used to obtain the highest classification accuracy of the feature subset. The parameters of the SVM classifier use a kernel function. The penalty parameter C and RBF parameters are selected by the Grid Search method.

Compared with other methods, the ten-fold cross-validation method is the best method of estimating the classification performance [42]. Therefore, the above-mentioned algorithm is tested with a 10-fold cross-validation method.

### Experimental results and analysis

Seven data sets and five algorithms were used in this experiment. After each algorithm runs 10 times on each data set, the maximum value of the classification accuracy and the length of the feature subset are displayed in Table 6. The average of the classification accuracy and the average of the feature subset length are shown in Table 7.

**Table 6:** The highest classification accuracy of each algorithm on each data set and its feature subset length.

Abbreviation	mrmr+PSO		mrmr+BBA		mrmr+CS		mrmr+GA		BGWO		MKMDIGWO	
	ACC	len	ACC	len	ACC	len	ACC	len	ACC	len	ACC	len
11t	92.0	48	72.3	50	90.8	**33**	91.9	58	92.2	506	**92.5**	38
BD	98.2	10	92.8	16	98.4	17	98.2	13	98.1	18	**98.4**	**10**
BP	86.3	17	70.7	19	86.3	21	85.3	24	87.4	17	**87.8**	**12**
DE	99.5	17	99.2	50	99.7	18	99.7	25	99.6	63	**99.7**	**4**
GE	77.3	15	69.1	12	77.9	16	77.3	16	78.3	13	**78.6**	**13**
LU	98.5	34	90.1	46	98.5	31	98.6	49	98.6	529	**100.0**	**25**
SH	85.4	20	73.4	24	85.8	28	83.9	20	86.5	23	**86.9**	**17**
EC	93.3	6	93.4	6	93.4	6	92.0	4	92.5	4	**93.4**	**4**
PA	98.5	9	98.5	13	98.5	13	97.5	17	98.5	11	**99.0**	**8**
PT	100.0	23	100.0	43	98.2	24	98.1	46	99.1	371	**100.0**	**13**
SC	61.0	62	73.8	75	68.33	62	64.2	62	73.3	4766	**74.8**	**62**
WI	100.0	9	100.0	9	100.0	8	99.4	10	100.0	9	**100.0**	**8**

**Table 7:** Average classification accuracy and average feature subset length of each of the six algorithms on each data set.

Abbreviation	mrmr+PSO		mrmr+BBA		mrmr+CS		mrmr+GA		BGWO		MKMDIGWO	
	ACC	len	ACC	len	ACC	len	ACC	len	ACC	Len	ACC	len
11t	91.5	42.0	69.0	42.6	90.2	**32.3**	90.4	53.6	91.2	498.6	**92.1**	38.0
BD	98.2	10.7	90.9	11.2	97.5	18.1	98.2	16.8	98.1	12.6	**98.3**	**10.0**
BP	84.5	16.1	68.4	17.2	85.7	20.1	83.6	19.4	86.6	17.6	**86.6**	**15.6**
DE	99.5	19.7	98.6	50.8	99.7	26.9	99.6	25.3	99.7	220.0	**99.7**	**5.6**
GE	77.1	14.4	68.4	**12.0**	77.9	16.6	77.2	14.8	78.1	12.4	**78.2**	13.3
LU	98.1	28.5	88.7	47.8	97.5	29.0	97.8	50.8	97.9	698.1	**98.3**	**28.1**
SH	84.5	18.3	72.6	21.9	85.0	26.5	82.8	22.2	84.9	18.8	**85.7**	**18.0**
EC	92.9	5.3	92.8	6.5	93.0	6.0	91.6	5.5	92.1	5.3	**93.4**	**5.0**
PA	98.0	9.9	98.2	11.8	98.2	11.7	97.2	9.8	98.3	11.5	**98.3**	**8.5**
PT	99.3	24.8	99.0	50.0	98.2	26.6	97.2	50.9	98.7	815	**100.0**	**16.9**
SC	58.5	57.0	72.2	73.2	68.4	56.0	61.5	60.0	73.7	6674	**73.8**	**54.0**
WI	99.6	8.6	99.6	8.8	100.0	8.8	98.9	8.7	99.9	10.1	**100.0**	**8.5**

From the above two tables, we can find that the MKMDIGWO algorithm achieves a high average classification accuracy on 12 data sets. On the LU, PT, WI dataset, the classification accuracy of the MKMDIGWO algorithm reached 100%. On the LU and SC data sets, the MKMDIGWO algorithm is at least 1% higher than other algorithms. In addition to the 11t and GE data sets, the MKMDIGWO algorithm has the shortest feature subset length on other algorithms. Especially on the DE and PT dataset, the MKMDIGWO algorithm’s dimensionality reduction effect is very obvious. The BGWO algorithm is a wrapper algorithm. Therefore, the effect of dimensionality reduction on high-dimensional data sets (11t, LU, PT, SC) is not obvious.

**Fig 11:**
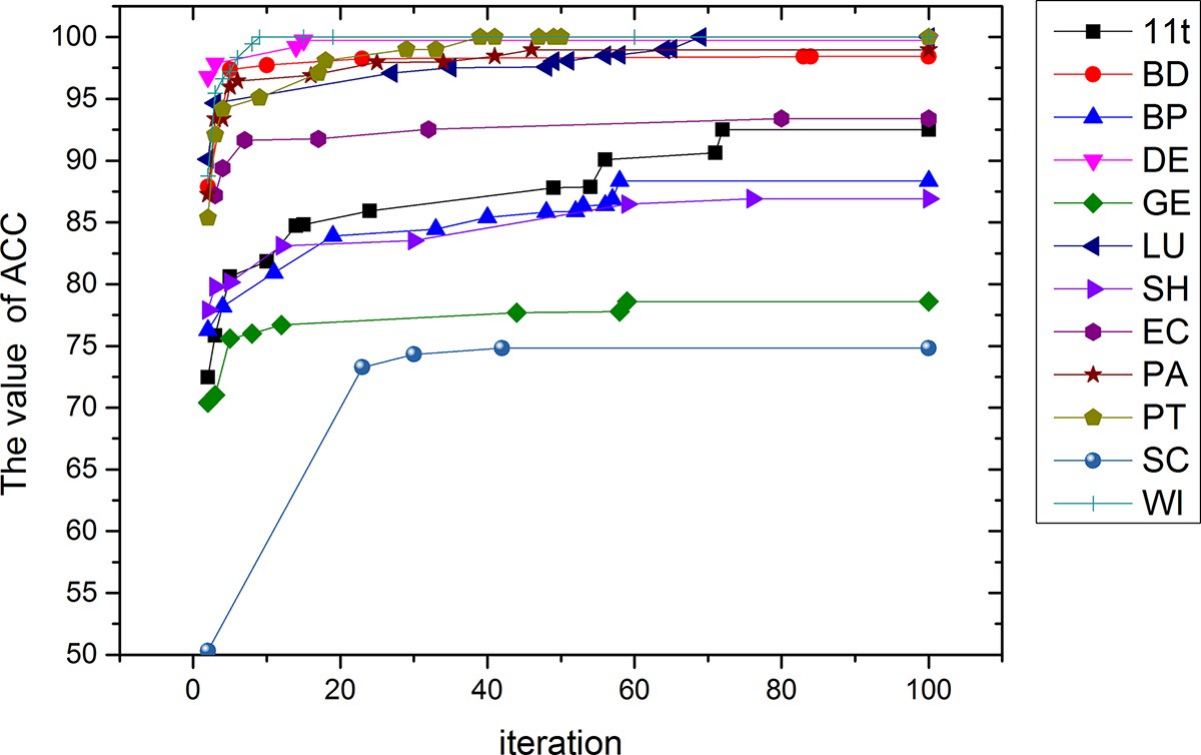
Classification accuracy of 100 iterations over 12 data sets.

From Fig 11, it can be seen that the abscissa is the number of iterations, from 0 to 100, and the ordinate is the classification accuracy of each data set. The classification accuracy rate on the six data sets of DE, BD, LU, PA, PT and WI has reached more than 98%, and the accuracy rate is relatively high. The classification accuracy rate of the 11t, BP, EC and SH data sets reached more than 85%. Only the classification accuracy of the GE and SC data set reached 78% and 74%, which is lower than 80%.

The highest classification accuracy obtained for each data set is formed by a combination of multiple filter algorithms and optimization algorithms. In the filter algorithm, the important features appear in the candidate feature subsets by the Kendall coefficient and the Euclidean distance measurement method. In the wrapper algorithm, after several optimizations, the feature subset with higher classification accuracy is found. And the best results have been found before reaching 100 iterations.

**Fig 12:**
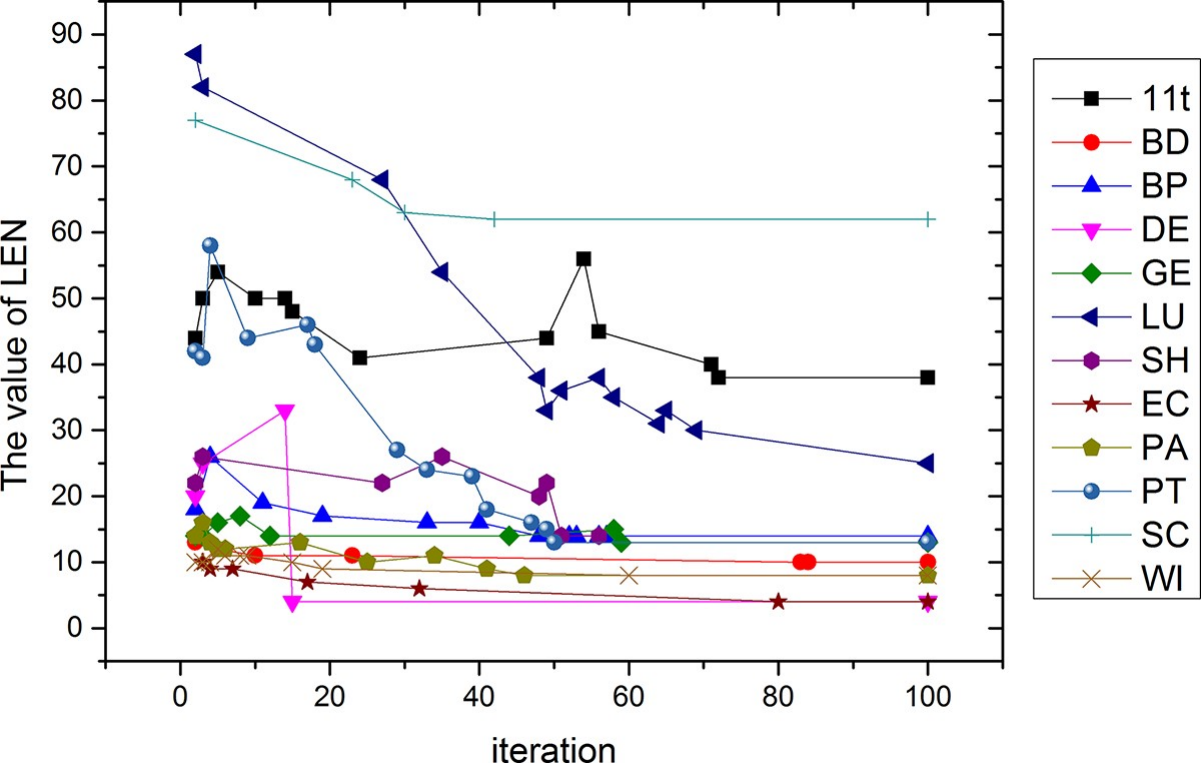
Feature subset length for 100 iterations over 12 data sets.

The Fig 12 show that the abscissa is the number of iterations, from 0 to 100, and the ordinate is the change in the length of the feature subset of each data set. The number of feature subsets on the three data sets of PT, LU, 11t, and SC is relatively small compared to the total number of features, reaching one thousandth, two thousandths, three thousandths and three thousandths of the total number of features respectively. On the DE, EC, BD, PA, BP, and SH datasets, the number of feature subsets exceeds 30% of the total number of features. Only the dimensionality reduction on the GE and WI data sets is more than 50%. Therefore, the proposed algorithm has a more significant dimensionality reduction effect on high-dimensional data sets.

**Fig 13:**
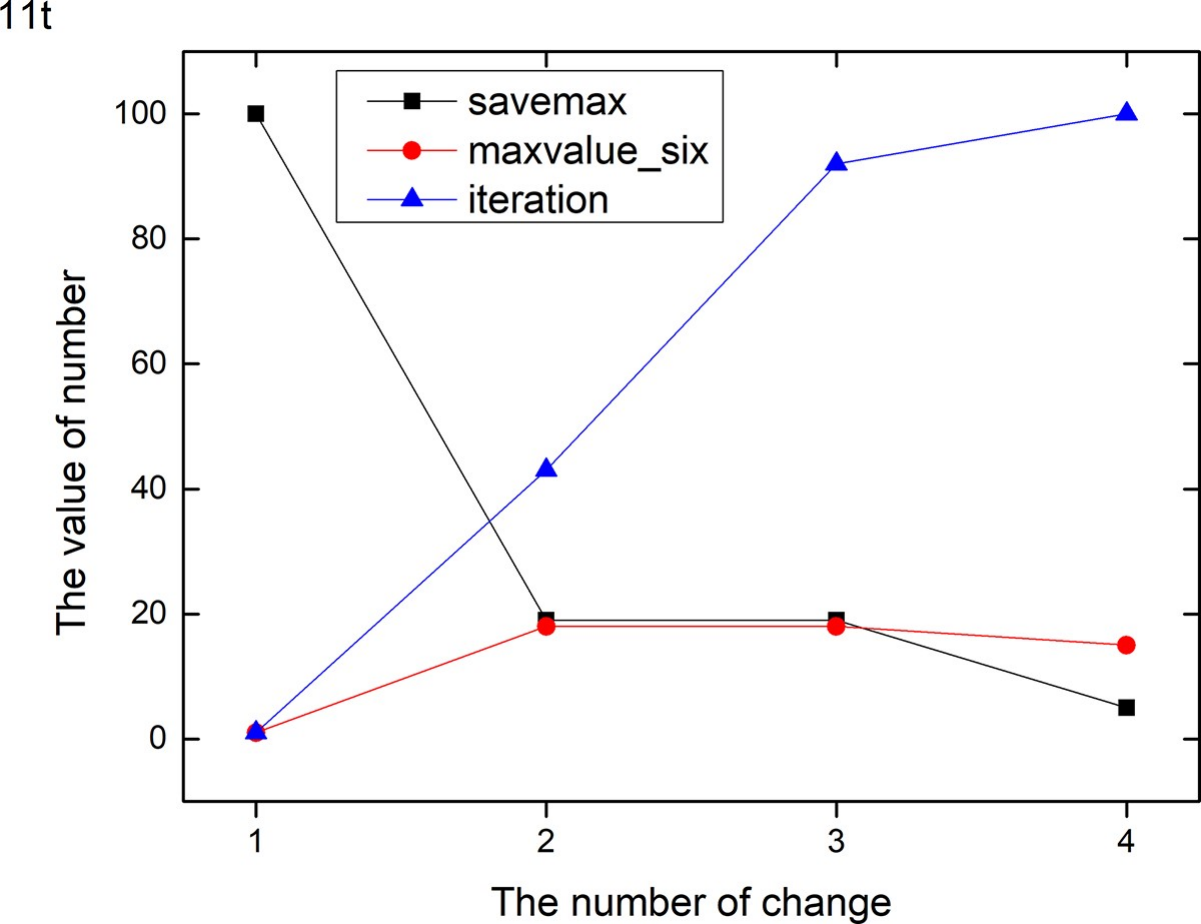
Modulation of damping oscillation on the 11t data set.

**Fig 14:**
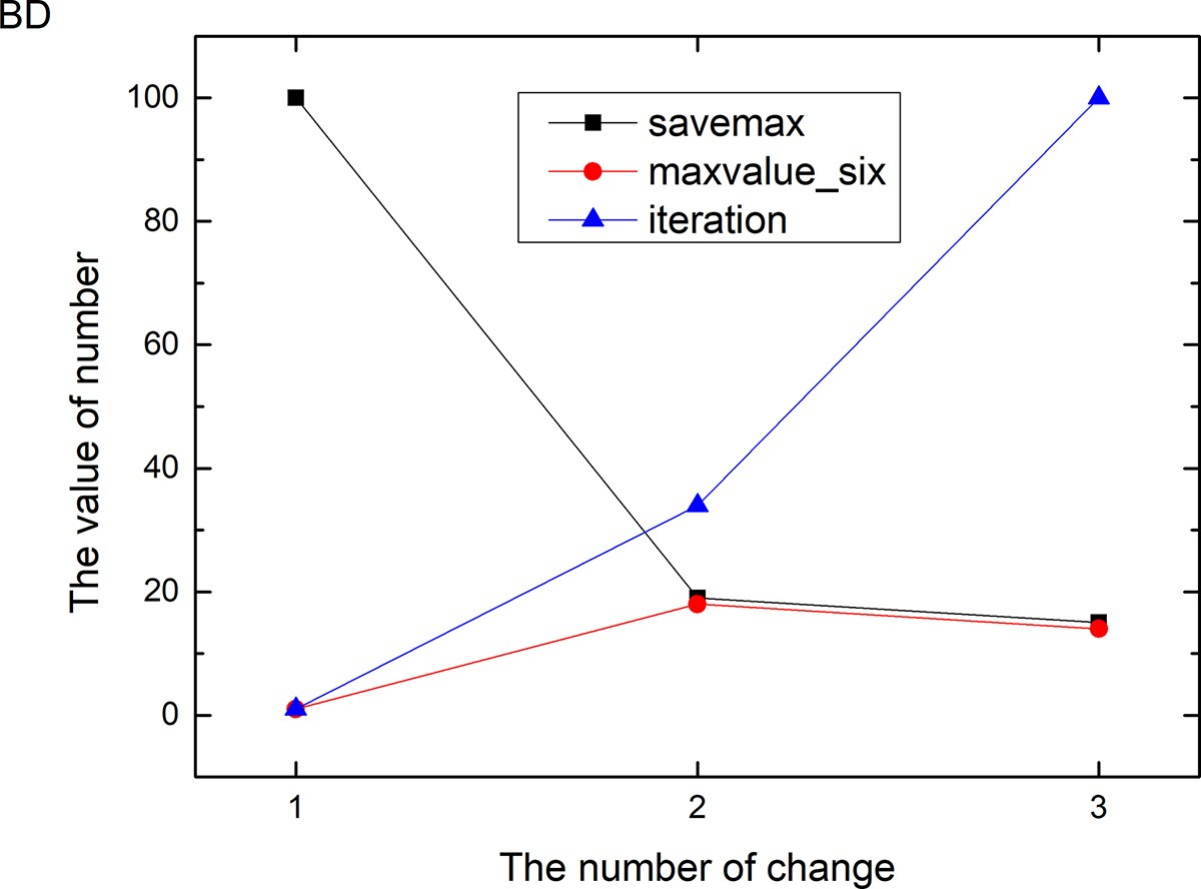
Modulation of damping oscillation on the BD data set.

**Fig 15:**
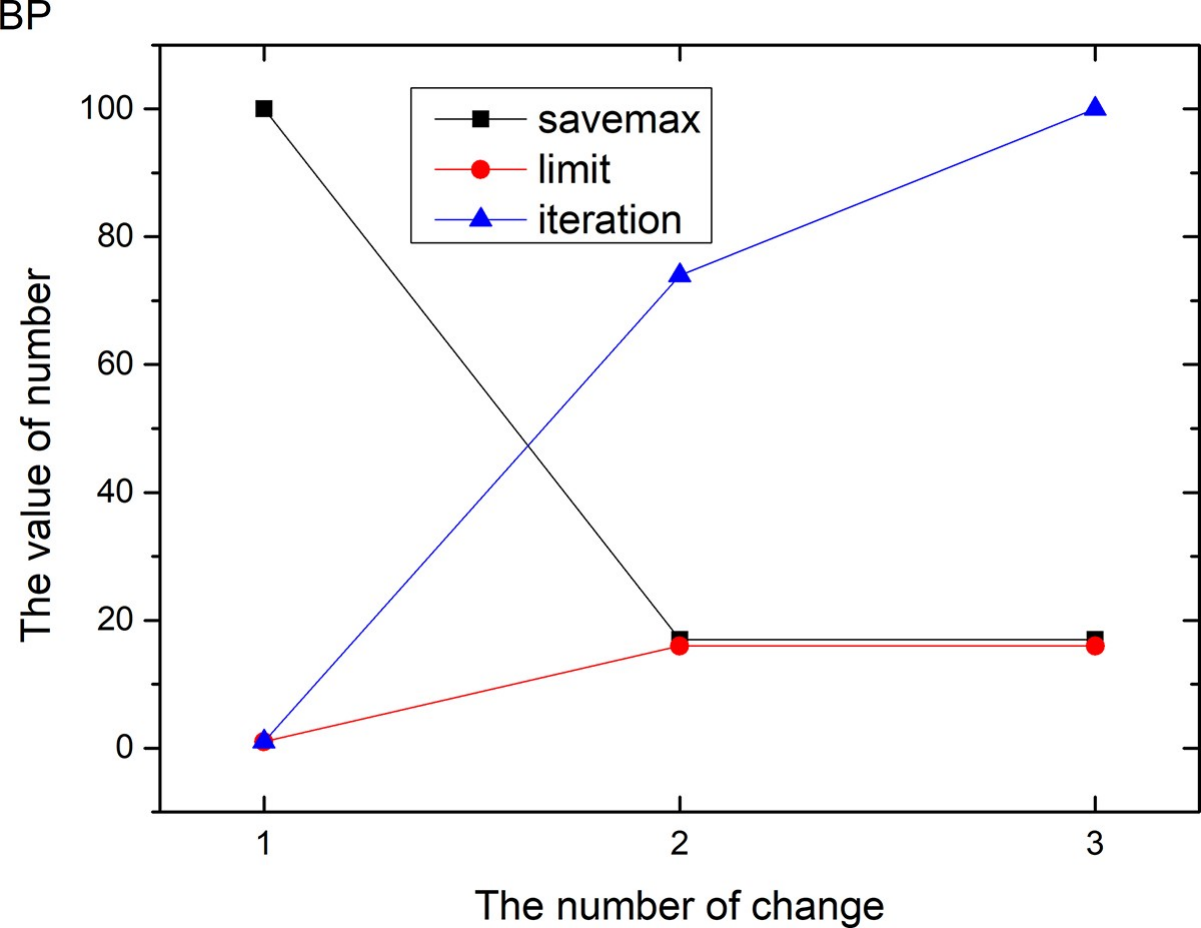
Modulation of damping oscillation on the BP data set.

**Fig 16:**
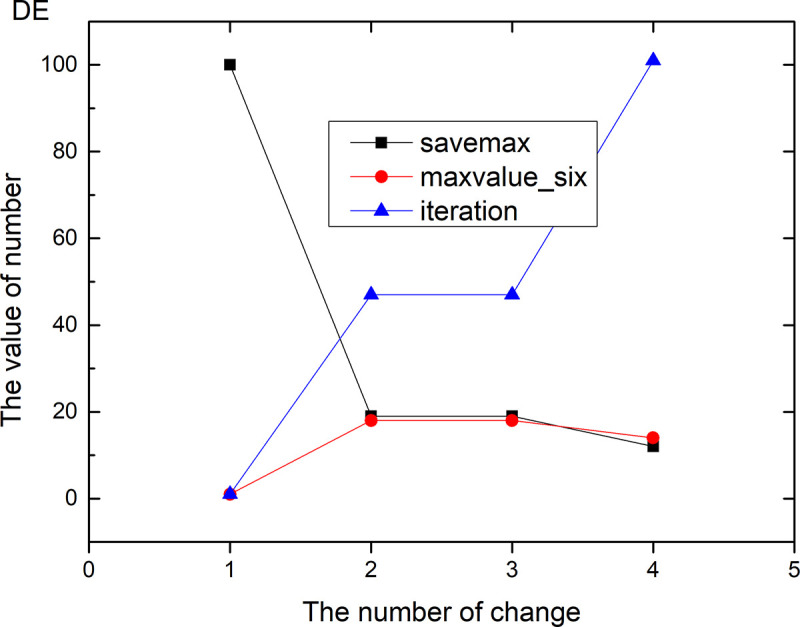
Modulation of damping oscillation on the DE data set.

**Fig 17:**
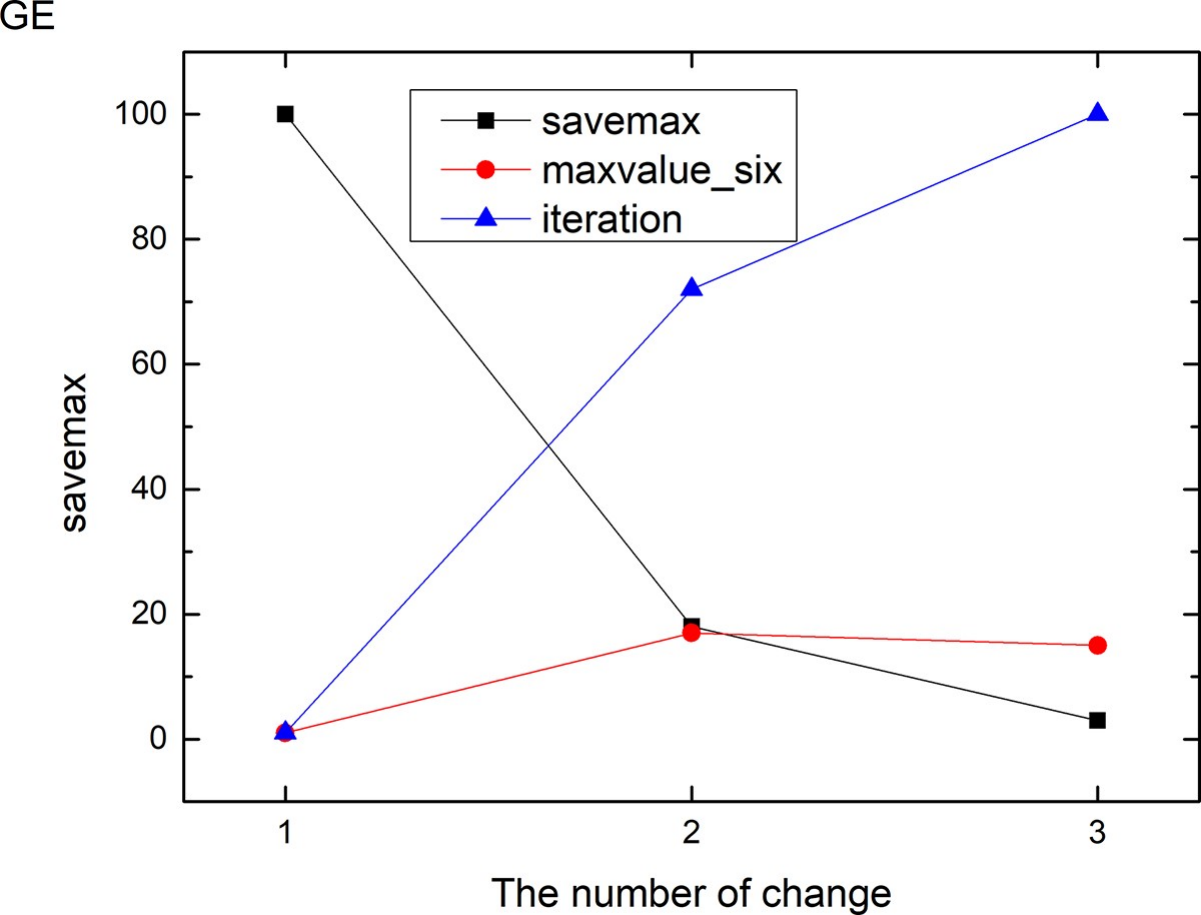
Modulation of damping oscillation on the GE data set.

**Fig 18:**
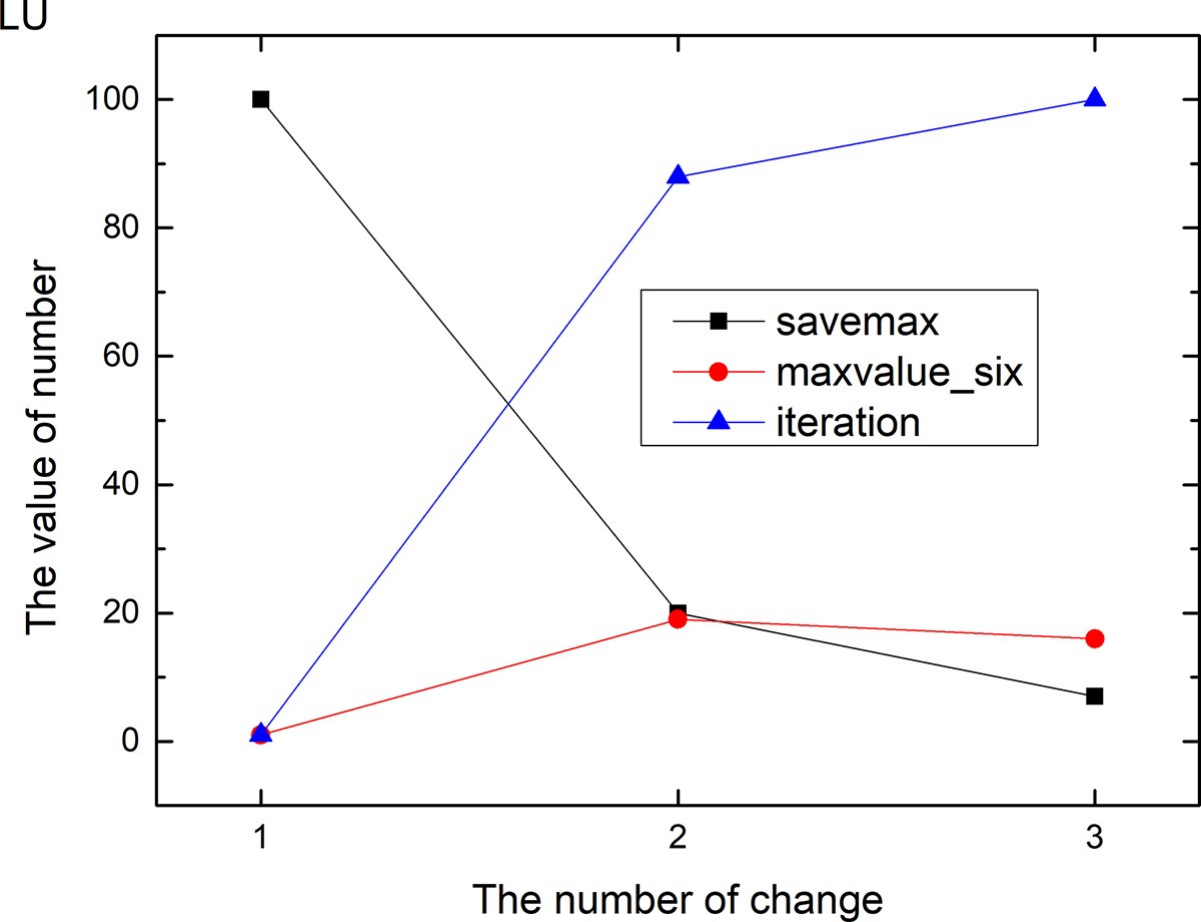
Modulation of damping oscillation on the LU data set.

**Fig 19:**
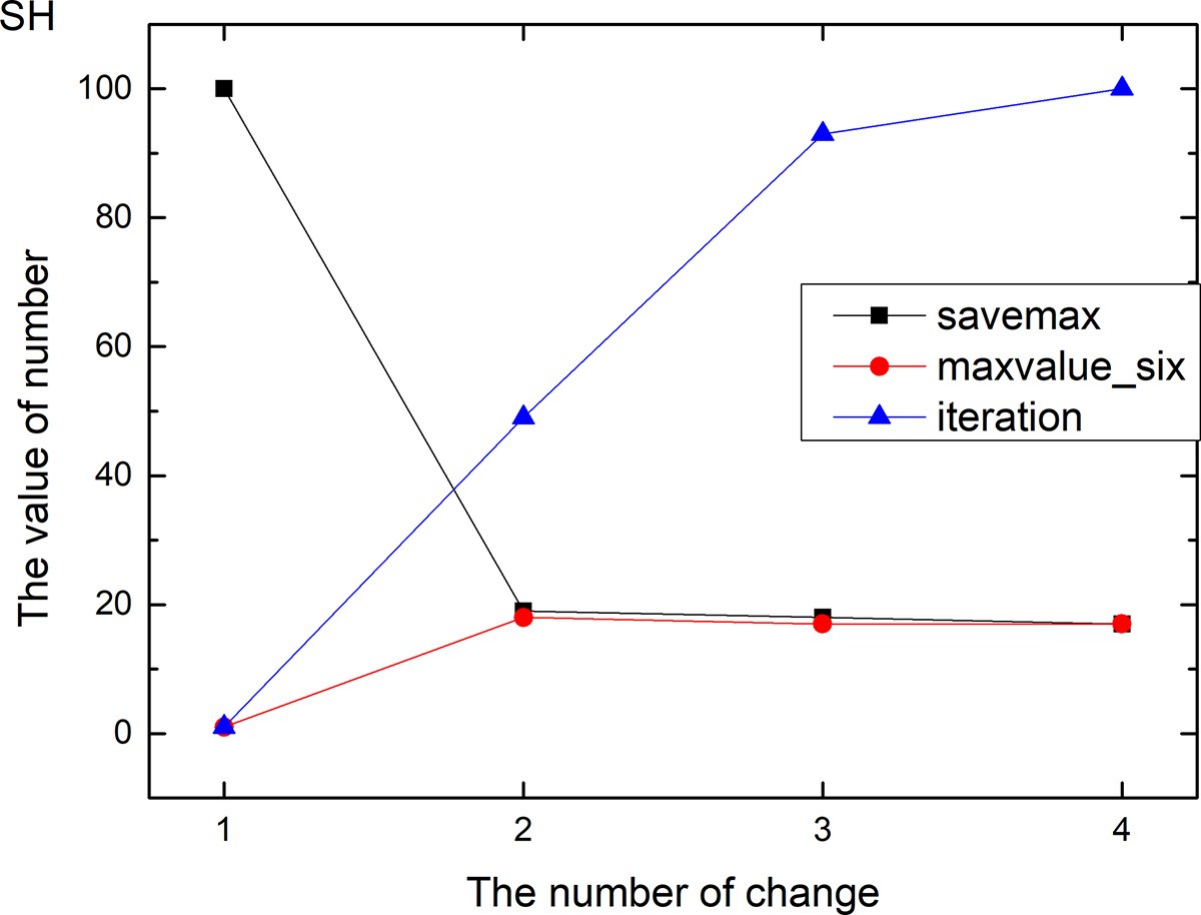
Modulation of damping oscillation on the SH data set.

**Fig 20:**
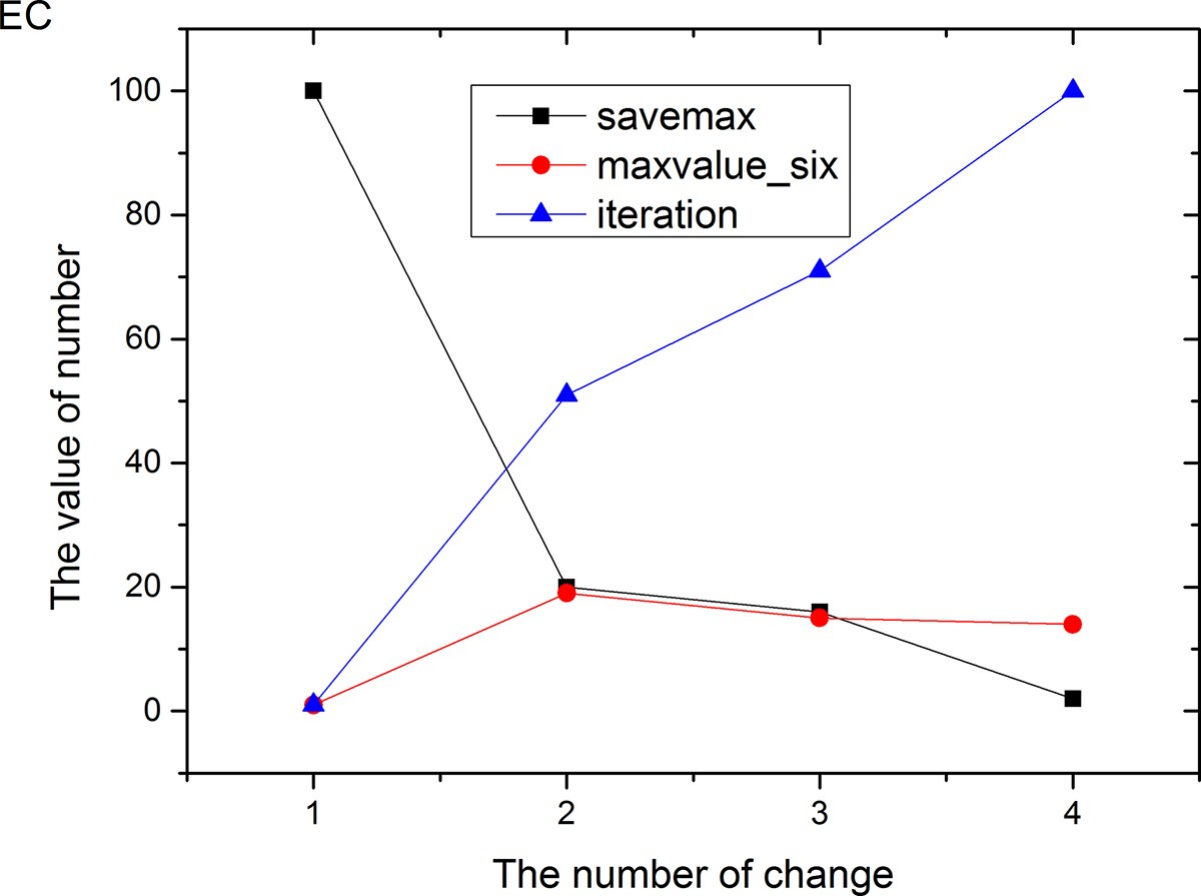
Modulation of damping oscillation on the EC data set.

**Fig 21:**
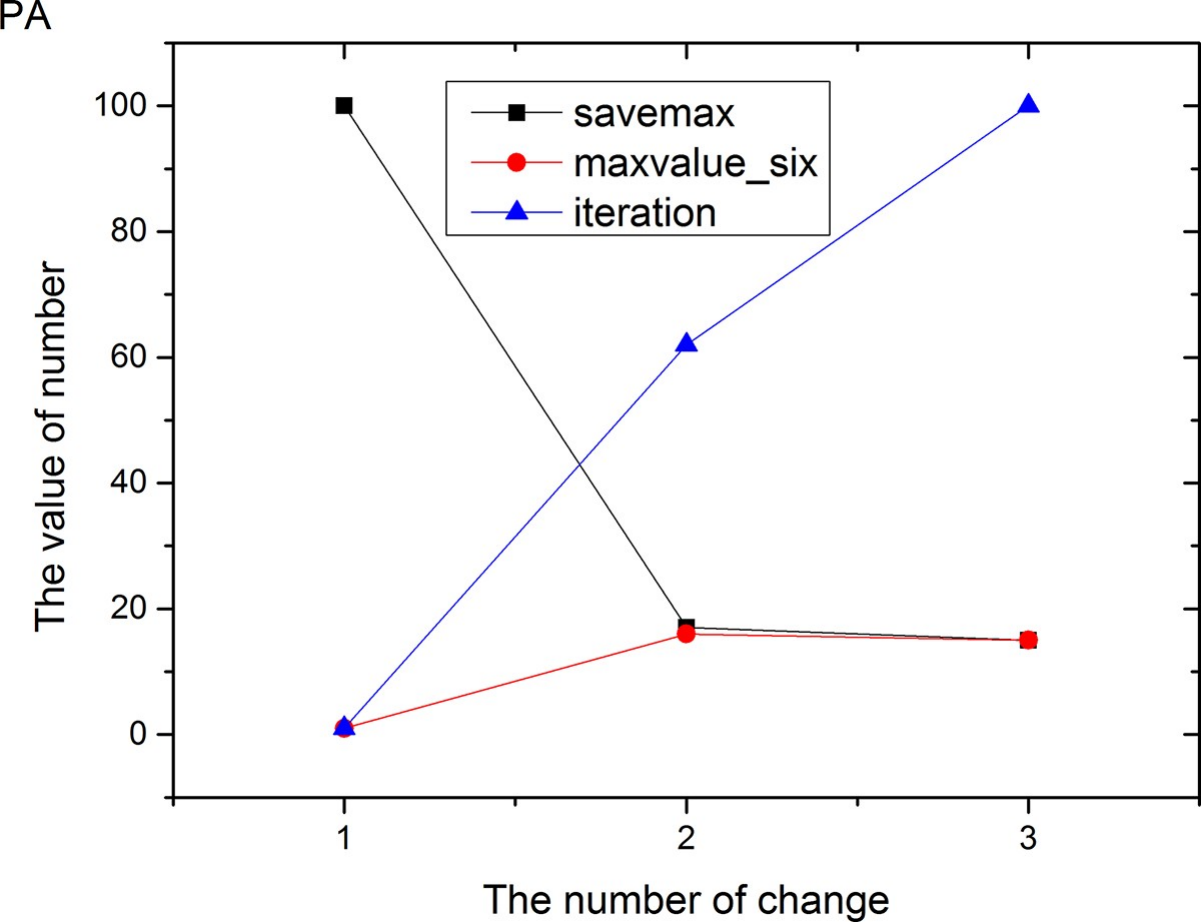
Modulation of damping oscillation on the PA data set.

**Fig 22:**
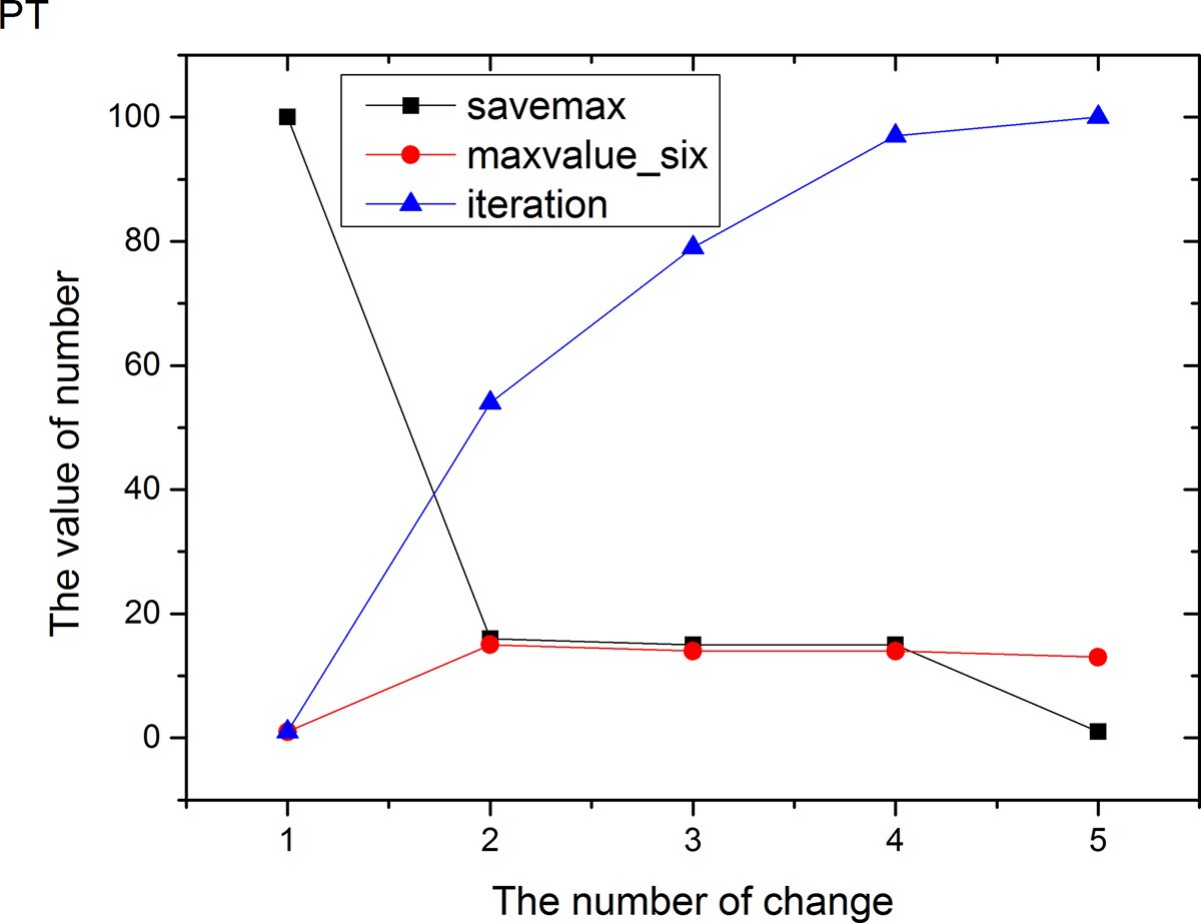
Modulation of damping oscillation on the PT data set.

**Fig 23:**
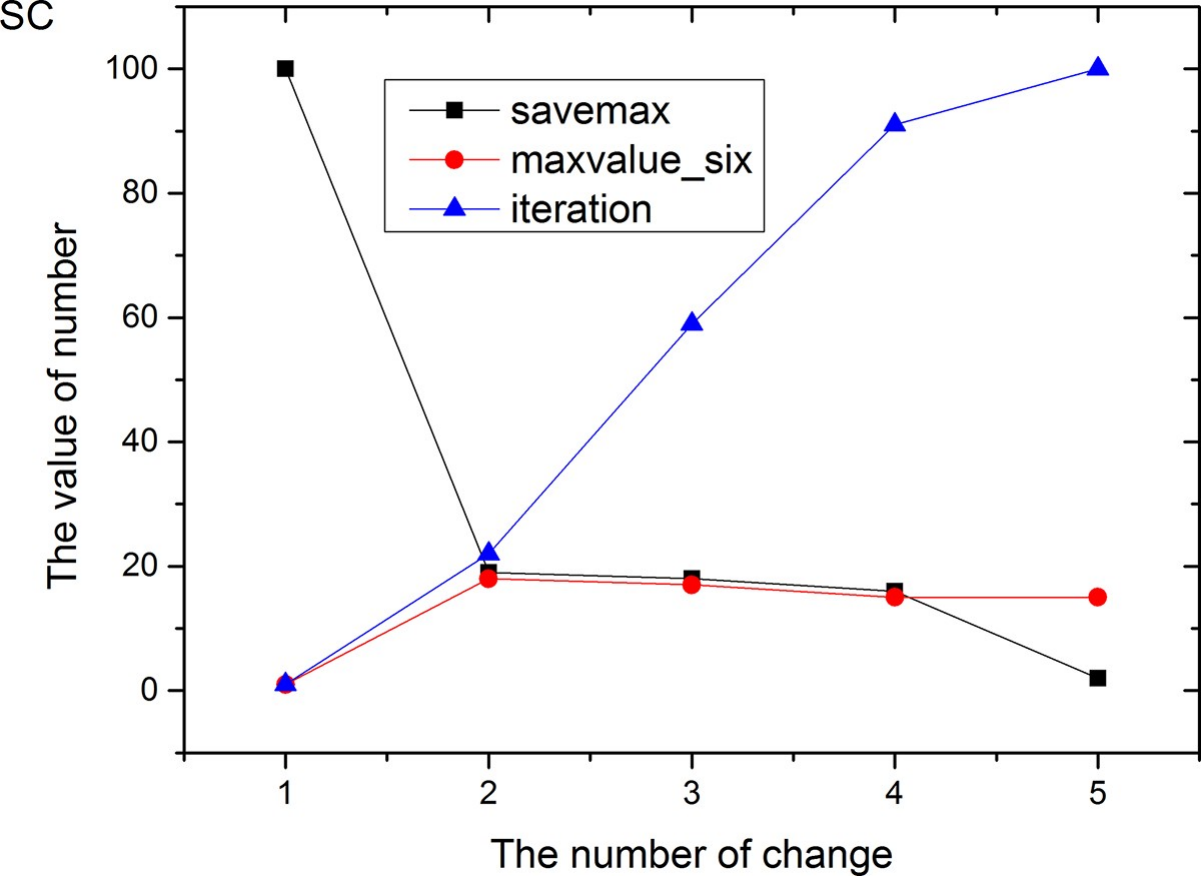
Modulation of damping oscillation on the SC data set.

**Fig 24:**
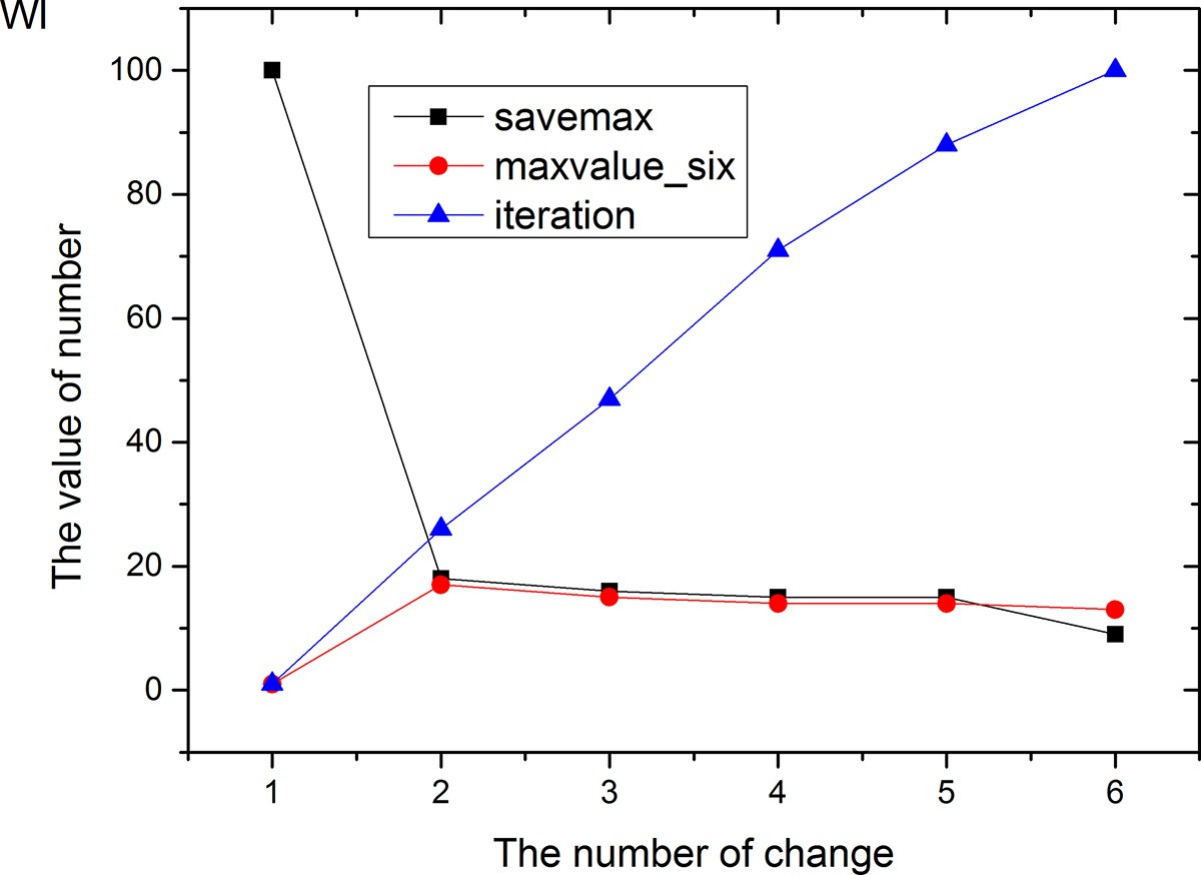
Modulation of damping oscillation on the WI data set.

Figs 13–24 tell us that the abscissa indicates the number of the most used values of the damping oscillation function, and the ordinate indicates the value of savemax. On the 12 data sets, the value of maxvalue_six represents the effect of different damping functions. The red line indicates the value calculated by the damping oscillating function, the black line indicates the number of times each maximum value is retained, and the blue line indicates the number of iterations when the damping oscillation is adjusted. The figure show that the black line is always slightly larger than the red line, that is, when the maximum number of retention times is just greater than the value of the damping oscillation function, the filter algorithm is executed.

In Damping Oscillation Adjustment, we mention the maximum value of the six damping oscillator functions. However, the twelve graphs show that the 11t, DE, EC, Lu and SH data sets use the four highest values, and three maximum values are used in BD, BP, GE and PA data sets. PT and SC dataset reach at five highest values. The WI data set is the most, it reaches 6 highest values. The sum of the maximum values of the damping oscillating functions in each graph is definitely less than 100. The number of iterations did not reach 100. During the iteration, as long as the value of ACC changes, the maximum number of reservations is assigned a value of 1. Therefore, the maximum number of damping oscillating functions in the twelve graphs is less than 7.

The Wilcoxon signed-rank test is proposed by Frank Wilcoxon as a non-parametric statistical hypothesis test [43]. This strategy is applied to contrasting two related samples. We can decide whether the corresponding data distributions are identical based on this test. In this paper, the Wilcoxon signed rank test is executed by SPSS software. The data information in Table 8 are the result of applying SPSS software. In Table 8, five pairs of Wilcoxon signed-rank tests are made on SC data set. With the significant level 0.05, it can be seen from Table 6 that the performance of MKMDIGWO is better than other five algorithms. In other words, the classification accuracy of each time is improved.

**Table 8:** The comparison based on Wilcoxon signed-rank test on SC data set.

Algorithms1	MKMDIWGO	MKMDIWGO	MKMDIWGO	MKMDIWGO	MKMDIWGO
Algorithms2	mRMR+PSO	mRMR+BBA	mRMR+GA	mRMR+CS	BGWO
Z	-2.805	-2.296	-2.810	-2.670	-1.989
P	0.005	0.022	0.005	0.008	0.047

## Conclusion

This paper proposes a new feature selection algorithm named MKMDIGWO, which has two characteristics. The first one is that the filter algorithm MKMD was proposed as the feature to score and sort, taking the feature with higher score as the candidate feature subset. The second is the combination of damping oscillation function adjustment filter algorithm and wrapper algorithm. In the MKMD algorithm, two parameters are introduced, which improves the correlation of candidate feature subsets and reduces their redundancy, and thus the classification accuracy is improved.

The advantages of filter methods and wrapper method feature selection are combined in MKMDIGWO. In the filter algorithm, two parameters are used to reduce the overall redundancy. Due to the adjustment of the maximum value of the damping oscillator function, the filter algorithm and the optimization algorithm are closely combined. Therefore, the solution quality of the feature selection problem is improved. It is shown in the experimental results that the proposed MKMDIGWO algorithm is much better than the other five algorithms.

This paper mainly discusses feature selection methods. The dimensionality of the data set used in the experiment is not high. In order to make the feature selection method more effective, in future work, we will use the feature selection method on high-dimensional data sets.
